# High Diversity of Novel Viruses in the Tree Pathogen *Phytophthora castaneae* Revealed by High-Throughput Sequencing of Total and Small RNA

**DOI:** 10.3389/fmicb.2022.911474

**Published:** 2022-06-16

**Authors:** Milica Raco, Eeva J. Vainio, Suvi Sutela, Aleš Eichmeier, Eliška Hakalová, Thomas Jung, Leticia Botella

**Affiliations:** ^1^Phytophthora Research Centre, Department of Forest Protection and Wildlife Management, Faculty of Forestry and Wood Technology, Mendel University in Brno, Brno, Czechia; ^2^Natural Resources Institute Finland (Luke), Helsinki, Finland; ^3^Mendeleum-Institute of Genetics, Faculty of Horticulture, Mendel University in Brno, Brno, Czechia

**Keywords:** mycovirus, dsRNA, RNA interference, multiple viral infections, forest pathogen, RdRp, ssRNA, oomycetes

## Abstract

*Phytophthora castaneae*, an oomycete pathogen causing root and trunk rot of different tree species in Asia, was shown to harbor a rich diversity of novel viruses from different families. Four *P. castaneae* isolates collected from *Chamaecyparis hodginsii* in a semi-natural montane forest site in Vietnam were investigated for viral presence by traditional and next-generation sequencing (NGS) techniques, i.e., double-stranded RNA (dsRNA) extraction and high-throughput sequencing (HTS) of small RNAs (sRNAs) and total RNA. Genome organization, sequence similarity, and phylogenetic analyses indicated that the viruses were related to members of the order *Bunyavirales* and families *Endornaviridae*, *Megabirnaviridae*, *Narnaviridae*, *Totiviridae,* and the proposed family “Fusagraviridae.” The study describes six novel viruses: Phytophthora castaneae RNA virus 1–5 (PcaRV1-5) and Phytophthora castaneae negative-stranded RNA virus 1 (PcaNSRV1). All six viruses were detected by sRNA sequencing, which demonstrates an active RNA interference (RNAi) system targeting viruses in *P. castaneae*. To our knowledge, this is the first report of viruses in *P. castaneae* and the whole *Phytophthora* major Clade 5, as well as of the activity of an RNAi mechanism targeting viral genomes among Clade 5 species. PcaRV1 is the first megabirnavirus described in oomycetes and the genus *Phytophthora*.

## Introduction

*Phytophthora* spp. are oomycetes taxonomically grouped under the kingdom Straminipila (Heterokonta; Beakes et al., 2014; Thines and Choi, 2016). Although similar in habitat and morphology to filamentous fungi, they are phylogenetically more closely related to brown algae and diatoms. Ubiquitous in marine, freshwater, and terrestrial environments (Beakes et al., 2014), many *Phytophthora* spp. are major plant and forest pathogens causing substantial economic losses in agriculture, horticulture, and silviculture, along with environmental damage in natural ecosystems, thereby impacting biodiversity worldwide (Erwin and Ribeiro, 1996; Jung et al., 2018). *Phytophthora castaneae* Katsura & K. Uchida (≡ *P. katsurae*, nom. Illegit.) is a plant pathogen mostly known for causing trunk rot on Japanese chestnut (*Castanea crenata* Sieb. et Zucc.). It is a homothallic species native to Southeast and East Asia (Jung et al., 2017, 2020) with papillate sporangia, easily distinguished from related species by its ornamented oogonia. It resides in *Phytophthora* phylogenetic Clade 5, one of the smallest *Phytophthora* clades, which currently comprises four species, i.e., *P. agathidicida* B.S. Weir, Beever, Pennycook & Bellgard, *P. castaneae*, *P. cocois* B.S. Weir, Beever, Pennycook, Bellgard & J.Y. Uchida, *P. heveae* A.W. Thomps., and the as yet undescribed taxon *P.sp.* “*novaeguineae*” (Martin et al., 2014; Weir et al., 2015). *Phytophthora* species from Clade 5 are distributed in East and Southeast Asia, Papua New Guinea, Eastern Australia, New Zealand, Hawaii, and Central and South America (Erwin and Ribeiro, 1996; Bruce, 1999; Martin et al., 2014; Weir et al., 2015; Jung et al., 2017, 2020; Legeay et al., 2020).

Fungal viruses (mycoviruses) have been discovered in many major taxa of phytopathogenic fungi (Ghabrial et al., 2015). In oomycetes, an increasing number of viruses with double-stranded (ds) RNA, positive-sense (+) single-stranded (ss) RNA, and negative-sense (−) single-stranded (ss) RNA genomes have been reported over the last few years (Shiba et al., 2018, 2019; Cai et al., 2019a,b; Botella et al., 2020; Botella and Jung, 2021; Fukunishi et al., 2021; Poimala et al., 2021). However, many species have yet not been screened for the presence of viruses. Eight novel linear (−)ssRNA viruses putatively belonging to the order *Bunyavirales* were recently described in *Halophytophthora* (Botella et al., 2020), a primarily marine sister genus of *Phytophthora* (Maia et al., 2022). A metagenomics study revealed a rich (−)ssRNA, (+)ssRNA, and dsRNA virome in *Plasmopara viticola* (Berk. & M.A. Curtis) Berl. & De Toni-associated lesions (Chiapello et al., 2020); a (+)ssRNA virus has been described in *Pl. halstedii* (Farl.) Berl. & De Toni and *Sclerophthora macrospora* (Sacc.) Thirum., C.G. Shaw & Naras (Yokoi et al., 1999, 2003; Heller-Dohmen et al., 2011; Grasse and Spring, 2017). In the oomycete genus *Pythium*, dsRNA and virus-like particles were observed in Australian isolates of *Py. irregulare* Buisman (Gillings et al., 1993). In an isolate of the mycoparasitic *Globisporangium nunn* (Lifsh., Stangh. & R.E.D. Baker) Uzuhashi, Tojo & Kakish., formerly classified as *Py. nunn* Lifsh., Stangh. & R.E.D. Baker, Pythium nunn virus 1 (PnV1), a distinct member of the viral genus *Gammapartitivirus* in the family *Partitiviridae*, was described (Shiba et al., 2018). Viruses related to the fusarivirus [Pythium ultimum RNA virus 1 (PuRV1)] and the totivirus [Pythium ultimum RNA virus 2 (PuRV2)] were discovered from a Japanese isolate of the plant-parasitic oomycete *G. ultimum* (Trow) Uzuhashi, Tojo & Kakish. (Fukunishi et al., 2021). A toti-like virus, Pythium polare RNA virus 1 (PpRV1), was reported from the heterothallic oomycete species *Py. polare* Tojo, van West & Hoshino isolated from a moss in the Arctic together with Pythium polare RNA virus 2 (PpRV2) and Pythium polare bunya-like RNA virus 1 (PpBRV1; Sasai et al., 2018). Another toti-like virus closely related to PpRV1 was reported in Japanese isolates of *G. splendens* (Hans Braun) Uzuhashi, Tojo & Kakish., formerly *Py. splendens* Hans Braun, Pythium splendens RNA virus 1 (PsRV1; Shiba et al., 2019).

Until very recently, there were only a few viruses described in the genus *Phytophthora*, mainly from the notoriously devastating potato blight pathogen *P. infestans* (Mont.) de Bary (Cai et al., 2009, 2012, 2013, 2019b; Cai and Hillman, 2013). Phytophthora infestans RNA virus 1 (PiRV-1) and Phytophthora infestans RNA virus 2 (PiRV-2) seem to represent entirely novel viral families (Cai et al., 2009, 2019b; Cai and Hillman, 2013). PiRV-2 is found to stimulate sporangia production in its host, *P. infestans* (Cai et al., 2019a). Phytophthora infestans RNA virus 3 (PiRV-3; Cai et al., 2013) clusters with the newly proposed family “Fusagraviridae” (Wang et al., 2016). Phytophthora infestans RNA virus 4 (PiRV-4) is an unclassified narnavirus characterized in the same isolate as PiRV-3 (Cai et al., 2012). Members of alphaendornaviruses were found in isolates of *Phytophthora* taxon “douglasfir,” *P. ramorum* Werres, De Cock & Man in ‘t Veld (Kozlakidis et al., 2010) and in isolates of a *Phytophthora* pathogen of asparagus collected in Japan (Uchida et al., 2021). Recently, a number of novel viruses were identified in several other *Phytophthora* species (Botella and Jung, 2021; Poimala et al., 2021; Xu et al., 2022). Thirteen bunya-like and two toti-like viruses have been detected by HTS of total and small RNA (sRNA) in *P. condilina* T.I. Burgess from *Phytophthora* phylogenetic Clade 6a (Botella and Jung, 2021). In addition to a totivirus Phytophthora cactorum RNA virus 1 (PcRV1; Poimala and Vainio, 2020), seven novel viruses were recently reported from *P. cactorum*. This included three viruses having genome affinities to members of *Bunyavirales*, three alphaendornaviruses, and an usti-like virus (Poimala et al., 2021). In New Zealand, a novel unclassified dsRNA virus designated Phytophthora pluvialis RNA virus 1 (PplRV1) has been described from *P. pluvialis* Reeser, W. Sutton & E.M. Hansen (Xu et al., 2022).

Besides conventional methods, such as dsRNA extraction based on *CF*-11 cellulose affinity chromatography (Morris and Dodds, 1979) novel technologies, such as high-throughput sequencing (HTS), have enabled rapid expansion of knowledge in this field of virology. The screening of isolates by dsRNA extraction followed by purification and *CF*-11 chromatography is a traditional and relatively inexpensive first approach for viral detection, but it has certain limitations. Such a method was developed primarily to detect viruses with dsRNA genomes and, to a certain extent, is able to detect ssRNA viruses during their replication phase. As such, the method is less likely to detect DNA viruses. In addition, ssRNA viruses could potentially go undetected as the replication of intermediate dsRNA may not accumulate sufficiently during cell infection to allow detection. This is likely to be the case in *Heterobasidion parviporum* Niemelä & Korhonen infected by viruses from two (+)ssRNA virus families *Narnaviridae* and *Botourmiaviridae*, detected by HTS of total RNA depleted of rRNA, but not discovered earlier by extensive dsRNA screening (Sutela et al., 2021). Likewise, in three different species of *Armillaria* (Fr.) Staude, the presence of several ssRNA viruses has been revealed by HTS of total RNA, while no dsRNA elements were detected (Linnakoski et al., 2021). In addition, the dsRNA extraction technique does not allow characterization of the virome of samples containing a complex range of viruses, and viral full-length characterization is time consuming and requires additional costs (Nerva et al., 2016). In contrast, novel technologies such as HTS of sRNA and total RNA are widely used in detection of viruses, with HTS of total RNA being the dominant method nowadays for both detection and full-length genome characterization, despite having higher per sample cost. In order to direct the translational machinery of the host cell to produce viral proteins and ensure their survival, all viruses independent of their genome organization (DNA or RNA) need to express their genetic material as functional messenger RNAs (mRNAs) during the early infection stage. From genome to mRNA, viruses follow different pathways, which can be attributed to their type of genome (Rampersad and Tennant, 2018). Thus, sequencing of total RNA and sRNAs derived from it, potentially allows detection of all types of viruses, including those with DNA genomes (Nerva et al., 2016).

RNA interference (RNAi) is one of several pathways grouped under RNA silencing phenomena, defined as sequence-specific RNA degradation activated by the presence of long dsRNAs (Ketting, 2011; Svoboda, 2020). RNAi is a conserved mechanism used by many eukaryotic organisms, including animals, plants, fungi (Meister and Tuschl, 2004; Chang et al., 2012), and oomycetes (Mascia et al., 2019), for regulating the activity of gene expression. An important role of this biological pathway is to respond to non-self dsRNAs and mediate protective mechanisms against RNA viral infection. One of the first discoveries of RNA silencing pathways and the first experimental evidence of such activity in fungi was reported from the heterothallic filamentous fungus *Neurospora crassa* Shear & B.O. Dodge (Romano et al., 1992), which today serves as a model organism for studying RNA silencing. As RNA silencing is triggered by the occurrence of viral dsRNAs in the cell, virus-infected organisms in which this system is active are enriched for fragments of RNA representing small segments of viral genomes. HTS of sRNAs has been successfully used to detect viruses in plants and fungal pathogens (Kreuze et al., 2009; Vainio et al., 2015a; Donaire et al., 2016; Adalia et al., 2018; Massart et al., 2019; Kocanová et al., 2020; Botella and Jung, 2021).

The objectives of this study were primarily to identify and characterize viruses in *P. castaneae* combining traditional and next-generation sequencing (NGS) techniques, and, in addition, to examine whether *P. castaneae* has an active RNAi mechanism and processes viral RNAs into siRNAs as an act of defense. To the best of our knowledge, this is the first virome investigation of the tree pathogen *P. castaneae* or any species of *Phytophthora* phylogenetic Clade 5.

## Materials and Methods

### *Phytophthora* Isolates

Four isolates of *P. castaneae*, i.e., VN999, VN1004, VN1008, and VN1012, from the culture collection at Phytophthora Research Centre (PRC), Mendel University in Brno, originally isolated in 2017 from rhizosphere soil of four *Chamaecyparis hodginsii* (Dunn) Rushforth trees growing in a montane *Chamaecyparis*-*Quercus* forest stand in northern Vietnam close to Sapa (Jung et al., 2020), were selected for this study. The selected isolates were maintained on V8-juice agar [V8A; 100 ml/l V8 juice (Hermann Pfanner Getränke, Lauterach, Austria), 16 g/l of agar (Sigma-Aldrich, St. Louis, MO, United States), 2 g/l of CaCO_3_, and 900 ml/l of distilled water] at 20°C in the dark. For total RNA extraction, isolates were grown on carrot juice agar [CA; 100 ml/l carrot juice (Hermann Pfanner Getränke), 16 g/l of agar (Sigma-Aldrich), 2 g/l of CaCO_3_, and 900 ml/l of distilled water] covered with cellophane membrane (EJA08-100; Gel Company Inc., San Francisco, CA, United States). For dsRNA extraction, the isolates were grown in 100 ml broth containing 0.2 g of CaCO_3_, 10 ml of V8 juice, and 90 ml of distilled water. The Erlenmeyer flasks with the liquid cultures were incubated at 20°C and 120 RPMs for 7–10 days in a Benchtop Incubated Shaker (IST-3075R; Lab Companion; Seoul, Republic of Korea).

### Isolation of dsRNA

The four *P. castaneae* isolates were first examined for putative virus presence using a modified dsRNA extraction protocol (Morris and Dodds, 1979) as described by Tuomivirta et al. (2002). Approximately 2 g of mycelium (fresh weight) was collected in a 50 ml Falcon tube together with two 10 mm diameter stainless steel beads, submerged in liquid nitrogen, and homogenized by vortexing using a Vortex-Genie 2 (SI-0256; Scientific Industries, Inc., Bohemia, NY, United States) and Vertical High-Speed 50 ml Tube Holder (SI-V203; Scientific Industries, Inc., Bohemia, NY, United States). After homogenization, dsRNA extraction was performed as described by Tuomivirta et al. (2002). The obtained products were visualized in a 1.2% agarose gel under ultraviolet light.

### Isolation of Total RNA

After 7–15 days, approximately 50 mg of fresh mycelium was collected from the cellophane surface in a sterile 2 ml RNAse/DNAse free tube supplied with lysing matrix C (740813.50; MN Bead Tubes Type C; Macherey-Nagel; Düren, Germany). The tubes were then submerged in liquid nitrogen and vortexed using the Vortex-Genie 2 and high-speed bead tube holder until the mycelium was ground to a fine powder. Total RNA was isolated using the SPLIT RNA extraction kit (Lexogen, Vienna, Austria) following the protocol provided by the manufacturer, eluted in 50 μl of SB buffer (Lexogen), and stored at −80°C.

### High-Throughput Sequencing of sRNAs

Prior to library construction, the quantity of extracted RNA was measured by a Modulus^™^ Single Tube Turner BioSystems device (9200–000; Modulus^™^; Turner BioSystems Inc.; Sunnyvale, CA, United States). The integrity of total RNA was visually assessed by running it on a 1.2% agarose gel stained with SYBR^™^ Green I Nucleic Acid Gel Stain (S7563; Invitrogen^™^; Thermo Fisher Scientific, Waltham, MA, United States). The libraries were generated directly from total RNA using the TruSeq small RNA library preparation kit (Illumina, San Diego, CA, United States) according to the manufacturer’s instructions. After reverse transcription and library amplification, libraries were loaded onto an Agilent High Sensitivity DNA chip (Agilent High Sensitivity DNA Kit, Agilent Technologies, Inc., Santa Clara, CA, United States) according to the manufacturer’s instructions and run on an Agilent – 2100 Bioanalyzer, Automated Electrophoresis Instrument (Agilent Technologies). cDNA constructs were purified and loaded into a Novex 6% TBE polyacrylamide 15-well gel (Life Technologies, Thermo Fisher Scientific; Waltham, MA, United States) in 5X Novex^®^ TBE Running Buffer (Life Technologies, Thermo Fisher Scientific; diluted to 1X before the run) and ran on the XCell SureLock^®^ Mini-Cell electrophoresis unit (Life Technologies, Thermo Fisher Scientific). After the run, the gel was stained with GelRed^®^ Nucleic Acid Gel Stain (Biotium, Fremont, CA, United States) and viewed under UV light. The bands that correspond to the adapter-ligated constructs derived from the 22-nucleotides (nts) and 30-nts sRNA fragments [the region between 145 base pairs (bp) and 160 bp amplicons] were cut out using a sterile razor blade. Gel slices were placed into 0.5 ml gel breaker tubes. The target products were purified from PAGE using glycogen (Sigma-Aldrich, Steinheim, Germany), 3 M NaOAc (Sigma-Aldrich), and ethanol. The final product was checked for size, purity, and concentration and the pellet resuspended (to reach a concentration of 2 nm) in 15 μl of Tris–HCl 10 mm, pH 8.5.

The libraries were sequenced on an Illumina MiniSeq sequencer using a MiniSeq High Output Reagent kit (75-cycles, Illumina). Constructed and normalized libraries were diluted to 1 nm and denatured. The 500 μl of prepared 1.8 pM library was then loaded to the reagent cartridge’s MiniSeq High Output Reagent (75 cycles). Six-base indexes were used as they significantly reduce ligation bias and ensure accurate measurement of miRNA expression. The libraries were sequenced in a single-read mode, 1 × 36 bp for VN999 and VN1008, and 1 × 50 bp for VN1004 and VN1012.

### Stranded Total RNA Sequencing

Total RNA of *P. castaneae* isolate VN999 and *P. condilina* BD661 (Botella and Jung, 2021) was pooled together and treated with TURBO DNA-free^™^ Kit (AM1907; Invitrogen^™^, Thermo Fisher Scientific). RNA quantity and quality were measured by the Qubit^®^ 2.0 Fluorometer (Life Technologies, Thermo Fisher Scientific) and Tape Station 4200 (Agilent Technologies). Total RNA was sent to Macrogen Inc., the Republic of Korea, for RNA library construction and deep sequencing. Prior to the library preparation, ribosomal RNA (rRNA) was depleted using the Ribo-Zero rRNA Removal Kit (Human/Mouse/Rat). The library was prepared using an Illumina TruSeq^®^ Stranded Total RNA Sample Preparation Kit (Illumina) and sequenced in paired-end (2 × 101 nts) mode on a NovaSeq6000 (Illumina).

### Bioinformatics

#### High-Throughput Sequencing of sRNAs

The raw data quality was checked using the FastQC-0.10.1 program (Andrews, 2010). Adapters, low-quality reads, and too short reads were removed by the FASTX-Toolkit Clipper,^1^ stating the Q33 parameter. Individual reads were assembled using a set of algorithms known as Velvet (version 7.0.4; Zerbino and Birney, 2008). Reads below 17 nts were removed. The reads were assembled with Velvet k-mer lengths from 13 to 29. All the assemblies produced by Velvet were merged using the AssemblyAssembler1.3 script.^2^ The obtained contigs were aligned to non-redundant (nr) viral protein and nucleotide databases using BlastX and BlastN (NCBI BLAST+ 2.12.0). The BlastX search was limited to records that included “Viruses” (taxid:10239). All the contigs were imported to Geneious Prime^®^ 2019.0.4 bioinformatics software for molecular biology and sequence analysis.

#### Stranded Total RNA Sequencing

For the data processing of stranded total RNA-Seq reads, two approaches were used. For both, quality control of raw sequencing data was performed using the FastQC-0.10.1 program (Andrews, 2010). In the first approach, the data were cleaned of the host reads by assembling it to the host genome. Because of the unavailability of the *P. castaneae* genome, the reads were assembled to the genome of the most similar species from the same clade, *P. agathidicida* GCA_001314435.1_NZFS3772v2_genomic.fna.gz (Studholme et al., 2016), using the BWA software package and MEM algorithm (Li, 2013). In the second approach, the reads were used in *de novo* assembly directly after quality control. *De novo* assembly for both methods was performed by Trinity v2.6.5 (Grabherr et al., 2011), and all subsequent steps were the same for both data sets. Contigs obtained by Trinity were searched for similarities to a custom virus protein database in BlastX (BLASTX 2.10.0+) algorithm (Altschul et al., 1997) with an E value set to 10^−5^. All sequences showing significant similarity to known viruses were also aligned in the nucleotide database BlastN (NCBI^3^ BLAST+ 2.12.0) to exclude contigs potentially originating from the host. Potential protein encoding segments were detected with coding open reading frame (ORF) finder at NCBI website.^4^ In order to find putative viral contigs having low sequence similarity with the viral proteins deposited to GenBank, Trinity contigs originating from cleaned total RNA-Seq reads were examined and ORFan contigs were retrieved as follows. The *de novo* assembly of total RNA library was mapped to the sRNA library of isolate BD661 (Botella and Jung, 2021) as well as the putative virus sequences hosted by VN999 using Geneious 10.2.6 assembler with medium-low sensitivity. The contigs not mapped were selected and assembled next to the sRNA library of VN999 using the same settings. Of mapped contigs those over 800 nts in length were selected and run with BlastX against the NCBI nr database (E value 10e^−6^) and, subsequently, the contigs having no hits were examined further with blast searches and Geneious software.

#### Depth of Coverage

In order to calculate coverage depth for each virus, and to get information on the number of reads representing individual viruses, raw reads were mapped to the final contigs of each virus. The number of total RNA reads for each virus was calculated by Bowtie 2 v2.3.0 (Langmead and Salzberg, 2012). The number of sRNA reads for each virus was calculated by the alignment program Bowtie 1 v1.1.2 suitable for aligning the relatively short sequencing reads (Langmead et al., 2009). Both types of alignments were done in Geneious Prime^®^ 2020.2.3. For calculation of the coverage depth, the following formula was used as:


$$\frac{\mathrm{total}\;\mathrm{no}.\;\mathrm{of reads}\;{\left(\mathrm{sRNA}/\mathrm{total}\;\mathrm{RNA}\right)}^{*}\;\times \;\mathrm{average read length}}{\mathrm{virus genome length}}$$

^*^Total number of reads mapped to the final virus sequence.

### Detection and Validation of Viruses by RT-PCR Amplification and Sanger Sequencing

After 7–15 days of growth, approximately 100 mg of mycelium was collected and frozen in liquid nitrogen. Total RNA was isolated by RNAzol^®^ RT (Sigma-Aldrich, Steinheim, Germany) following the manufacturer’s recommendations with some modifications. The samples were homogenized in 1 ml of RNAzol^®^ RT (Sigma-Aldrich) using a Mixer Mill MM 400 (Retsch GmbH, Haan, Germany) for 2 min at 30 Hz. After the isopropanol precipitation step, the samples were loaded on RNA extraction columns (Lexogen, Vienna, Austria) and centrifuged at high speed for 30 s (RT). This step was repeated until the whole sample was loaded. The columns were then washed twice with 600 μl 75% ethanol and spin-dried after the last wash for at least 1 min at high speed. The columns were then transferred to sterile nuclease-free 1.5 ml tubes. The RNA was eluted by adding 50 μl Elution buffer SPLIT RNA extraction kit (Lexogen) preheated to 70°C, incubated for 1 min, and then centrifuged for 1 min. The quality of total RNA was assessed by visualization under UV light in a 1.2% agarose gel (visible presence of 18 S and 28 S rRNA bands). A Qubit^®^ 2.0 Fluorometer (Life Technologies, Thermo Fisher Scientific) was used to estimate RNA quantity. The final product was stored at −80°C. Prior to cDNA synthesis, total RNA was incubated for 10 min at 65°C. cDNA was synthesized using a High-Capacity cDNA Reverse Transcription Kit (Applied Biosciences, Park Ave, NY, United States). To confirm the successful synthesis of cDNA, the actin housekeeping gene was amplified with primers MIDFWACT and MIDREVACT (Weiland and Sundsbak, 2000). Every cDNA was checked, and if amplification was successful, the cDNA was used in a PCR reaction with virus-specific primers. The virus-specific primers were designed to amplify a fragment of the RNA-dependent RNA polymerase (RdRp) or capsid protein (CP) gene of putative viruses. All virus-specific primers were designed by Primer 3 2.3.7 in Geneious Prime^®^ 2020.2.3 (detailed information is presented in Supplementary Table 1). PCR amplification was performed with 20 μl Platinum^™^ II Hot-Start Green PCR Master Mix (2X; 14001014; Invitrogen^™^; Thermo Scientific) designed for universal primer annealing, 1 μl of each 10 mm primer, 3–5 μl of cDNA depending on the virus and its abundance and PCR grade water in a total volume of 50 μl. Cycling conditions followed the manufacturer’s recommendations, and the annealing temperature of 60°C was universal for all primers. Amplification was checked in 1.2% agarose gels stained with DNA Stain G (39803; SERVA, Heidelberg, Germany) after separation by electrophoresis (120 V, 50 min). All amplicons of the appropriate size were sent to Eurofins Genomics (Ebersberg, Germany) to be sequenced in both directions.

### Validation of Nearly Complete Viral Genomes and the Reference Sequences by Sanger Sequencing and Detection of Viral Strains

For verifying the accuracy of the final viral contigs obtained after the *de novo* assembly of total RNA reads and for determination of the exact nucleotide sequence of virus variants among studied isolates, virus-specific primers (Supplementary Table 2) covering almost entire genome length were designed and obtained amplicons were Sanger sequenced. Total RNA was extracted by RNAzol^®^ RT (Sigma-Aldrich) and transcribed to cDNA as described above. RT-PCR was conducted as described previously in 25 μl reaction volumes using 1.5–2.5 μl of cDNA. Obtained amplicons were sent to Eurofins Genomic to be Sanger sequenced in both directions. Pairwise sequence alignments were done by MAFFT v7.450 (Katoh and Standley, 2013) in Geneious Prime^®^ 2020.2.3. The accuracy of the Trinity contigs was validated in reference isolate VN999 by mapping the Sanger sequences obtained from VN999 viral amplicons to the final viral contigs by Geneious mapper using Medium-Low sensitivity settings.

### RNA Structures and Conserved Domains

NCBI CD-search tool^4^ (last accessed on 08.03.2022) was used for search of putative conserved domains. For the prediction of H-type pseudoknots in viral RNA sequences, DotKnot^5^ was used with maximum free energy (MFE). In order to look for putative conserved motifs of RdRp regions of putative viruses, predicted amino acid (aa) sequences were aligned using MUSCLE 3.8.425 (Edgar, 2004) in Geneious Prime^®^ 2020.2.3 to aa sequences of related viruses retrieved from the GeneBank. The % of similarities were calculated based on the Blosum62 score matrix with a threshold of 1.

### Phylogenetic Analyses

Amino acid (aa) sequences of RdRp regions of each virus were included in phylogenetic analyses. Sequences were aligned in Geneious 2020.2.3 by MUSCLE 3.8.425 (Edgar, 2004) together with known aa sequences of viruses considered to be related. Phylogenetic trees were built using the maximum likelihood method (Stamatakis et al., 2008) in RAxML-HPC v.8 on XSEDE running in the CIPRES Science Gateway web portal (Miller et al., 2010). Bootstrapping was performed by using the recommended parameters provided by the CIPRES Science Gateway portal. In order to avoid thorough optimization of the best-scoring ML tree at the end of the run, the GAMMA model was used. As the substitution model for proteins, the Jones–Taylor–Thornton (JTT) model was used. The trees were visualized in FIGTREE (V1.4.4).

## Results

### DsRNA Screening

Screening of possible viral dsRNA molecules of each of four Vietnamese isolates of *P. castaneae* (VN999, VN1004, VN1008, and VN1012) revealed different dsRNA banding patterns (Supplementary Figure 1). Fragments of *ca.* 7 and 0.9 kb were visible in all four isolates. In addition, bands of *ca.* 1.9 and *ca.* 0.5 and a band of *ca.* 10–20 kb could also be observed. In isolate VN999, an additional band of *ca.* 4 kb occurred. There was a lack in the consistency of the banding patterns, i.e., not all the bands appeared in every dsRNA extraction performed. As dsRNA occurrence indicates putative viral presence, all four isolates underwent HTS of sRNAs. Isolate VN999 was selected for stranded RNA sequencing.

### High-Throughput Sequencing, *de novo* Assembly, Size Profiling of sRNAs, and Identification of sRNA Contigs

In the first run, sequencing of three separately indexed libraries, VN1004, VN1012, and a third library that is not part of this study, gave 11,205,897 reads. 10,836,989 reads passed through the quality filtering stage; 33.34 and 33.59% of reads belonged to VN1004 and VN1012, respectively. The second run including libraries of isolates VN1008 and VN999 resulted in 19,467,986 reads, of which 18,094,637 passed quality filtering; 27.86 and 37.22% of reads belonged to VN999 and VN1008, respectively. The remaining reads from both runs belonged to libraries that are not part of this study.

**Figure 1:**
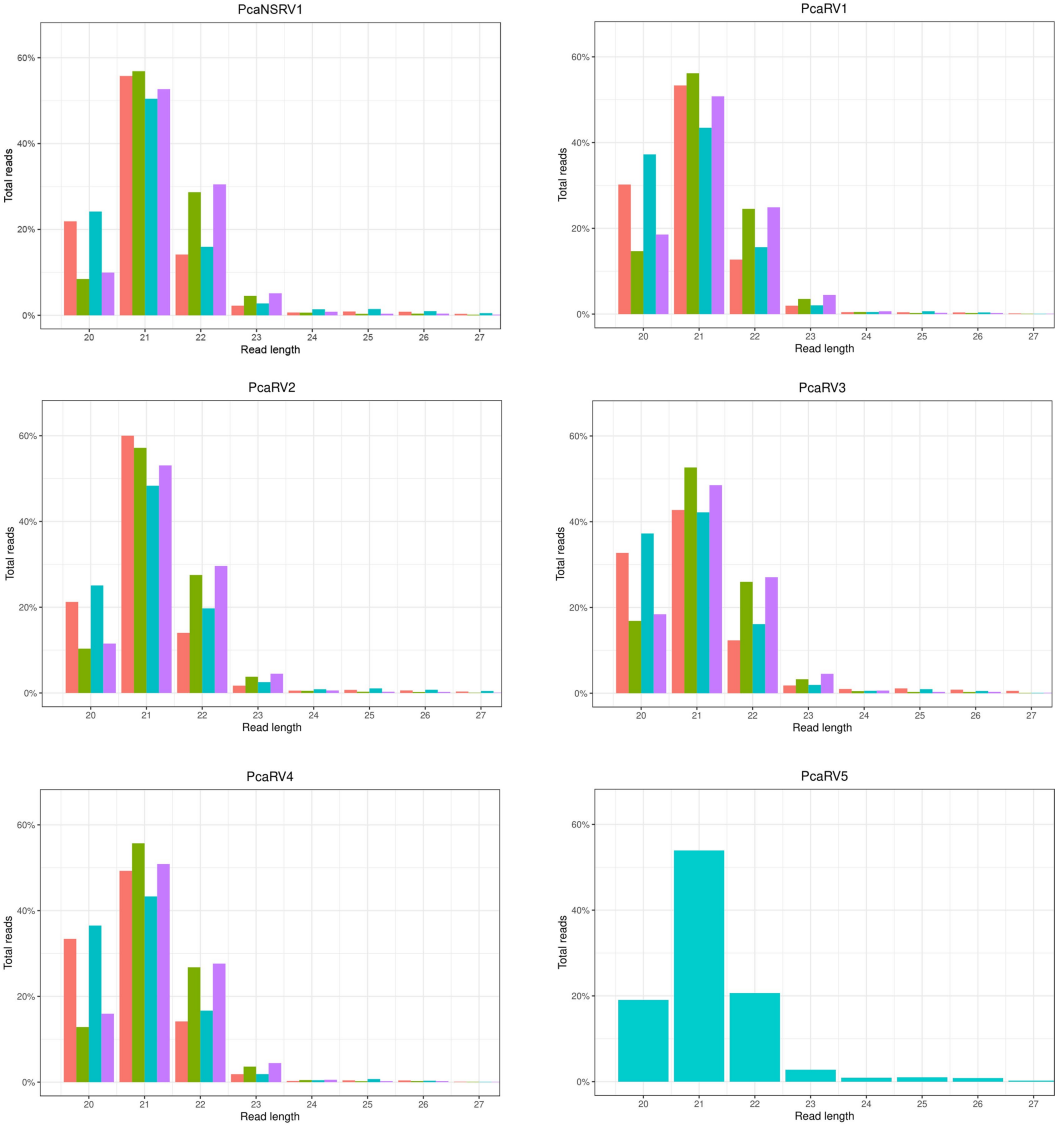
sRNA size distribution of raw VN999 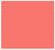,VN1004 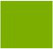, VN1008 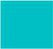, and VN1012 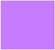 reads, respectively, after mapping to the final genomes of viruses PcaNSRV1 and PcaRV1-5, respectively. Mapping was done in Geneious Prime^®^ 2020.2.3 using Bowtie mapper. The final graphs were constructed in R Core Team (2020). Sizes of sRNA profiles starting from 28 nt that were represented with less than 1.5% of total reads per virus were omitted during graph preparation for a better visualization.

A graphical representation of the total number of raw sequences and sRNA size profiles per isolate are presented in Supplementary Figure 2. The number of individual contigs produced by *de novo* assembly, usage of assembly assembler script, and details of contigs length are presented in Supplementary Table 3. Virus derived sRNA profiles were obtained by mapping raw sRNA reads to the final viral contigs (Figure 1). A similar read length distribution was observed between the data sets and viruses. The highest proportion of reads were 21 nts in length while 20 and 22 nts peaks were also prominent (Figure 1).

The BlastX search revealed a high number of potential novel viruses from different families in each of the four isolates (Supplementary Tables 4–7). After excluding contigs that could represent the host genome with the aid of the BlastN search, 30 contigs of isolate VN999 (Supplementary Table 4), 74 contigs of VN1004 (Supplementary Table 5), 31 contig of VN1008 (Supplementary Table 6), and 61 contig of VN1012 (Supplementary Table 7) were selected based on their similarity to known viruses. Based on Blast homology searches, all of the four studied isolates were found to be infected by viruses sharing genomic affinities with viruses of the order *Bunyavirales* and families *Megabirnaviridae*, *Narnaviridae,* and *Totiviridae*. In addition, the BlastX search revealed contigs of the proposed virus family “Fusagraviridae” (Wang et al., 2016) in VN1004 and *Endornaviridae* in VN1008.

### Identification of Final Viral Sequences in Total RNA

Isolate VN999 was selected for carrying out stranded total RNA sequencing, which generated 127,332,406 reads. Raw data stats are displayed in Supplementary Table 8. *De novo* assembly of reads cleaned from the host produced 100,903 contigs with a minimum length of 201 nts and a maximum length of 21,824 nts. Blast comparison of the contigs revealed that isolate VN999 hosts several novel putative viruses with sequence similarities to known members of the order *Bunyavirales*, family *Megabirnaviridae*, family *Narnaviridae*, family *Totiviridae,* and the proposed family “Fusagraviridae” (Wang et al., 2016). Five viruses (one representative per viral family) from this data set were chosen for further characterization (Table 1). The accuracy of the final virus contigs was confirmed by Sanger sequencing of near full-length coding regions (Supplementary Table 9).

**Table 1:** Identification of the viruses found by total RNA seq in *P. castaneae* isolate VN999 and a virus found in VN1008 by sRNA seq and the most similar sequences in GenBank based on a BlastX search.

Virus name	Acronym^1^	Length^2^	GenBank^3^	Putative family/Order^4^	BlastX best hit^5^	E value	QC(%)^6^	I (%)^7^	Identified by BlastX search of Velvet contigs (yes/no)
Phytophthora castaneae RNA virus 1	PcaRV1	6,337	MZ269516	*Megabirnaviridae*	RdRp Charybdis toti-like virus (DAZ87259.1)	2e^−59^	34	29.08	yes
Phytophthora castaneae RNA virus 2	PcaRV2	2,891	MZ269517	*Narnaviridae*	RdRp Sanya narnavirus 11 (UHM27569.1)	2e^−102^	81	32.20	yes
Phytophthora castaneae RNA virus 3	PcaRV3	5,470	MZ269518	*Totiviridae*	putative RdRp polymerase Totiviridae sp. (UHS72506.1)	0.0	39	50.75	yes
Phytophthora castaneae RNA virus 4	PcaRV4	6,884	MZ269519	*“*Fusagraviridae*”*	ORF2 Bremia lactucae associated fusagravirus1 (QIP68010.1)	7e^−47^	31	28.15	no^*^
Phytophthora castaneae RNA virus 5	PcaRV5	2,182^x^	ON131873	*Endornaviridae*	hypothetical polyprotein Diatom colony associated dsRNA virus 15 (YP_009552081.1)	2e^−123^	99	34.82	yes
Phytophthora castaneae negative-stranded RNA virus 1	PcaNSRV1	8,345	MZ269515	*Bunyavirales*	polyprotein Phytophthora cactorum bunyavirus 1 (QUA12643.1)	0.0	97	57.16	yes

1 Acronym of Phytophthora castaneae RNA virus 1–5 and Phytophthora castaneae negative-stranded RNA virus 1.

2 Length, virus sequence length obtained through Trinity assembly.

3 GenBank accession number of each virus.

4 Putative Family/Order, placement of the viruses according to their genome organization and phylogenetic analysis.

5 The most similar virus according to BlastX search when the whole virus sequence is blasted against the (nr)protein sequence database in the online version of NCBI BlastX (date of the last search: 01.04.2022).

6 QC, Query cover.

7 I, Identity.

*PcaRV4 was identified by BlastX search of Velvet contigs only in one isolate (VN1012).

x Sequence of PcaRV5 is surely partial. The date of the last BlastX search 01.04.2022.

### The Six Novel Viruses Hosted by *Phytophthora castaneae* Isolates

The RT-PCR with virus-specific primers was utilized to confirm that the four *P. castaneae* isolates included in the sRNA analyses were infected with five putative virus species in addition to a sixth virus hosted by one of the *P. castaneae* isolates. The Trinity assembly of the total RNA library contained the complete coding virus sequences of the five putative viruses showing similarity with members of *Bunyavirales*, *Megabirnaviridae*, *Narnaviridae*, *Totiviridae,* and “Fusagraviridae” (Table 1). Furthermore, virus sequences having similarity with *Endornaviridae* were detected in the sRNA of VN1008, and a partial sequence of a novel endorna-like virus was obtained with Sanger sequencing (Table 1).

#### Megabirna-Like Virus

**Figure 2:**
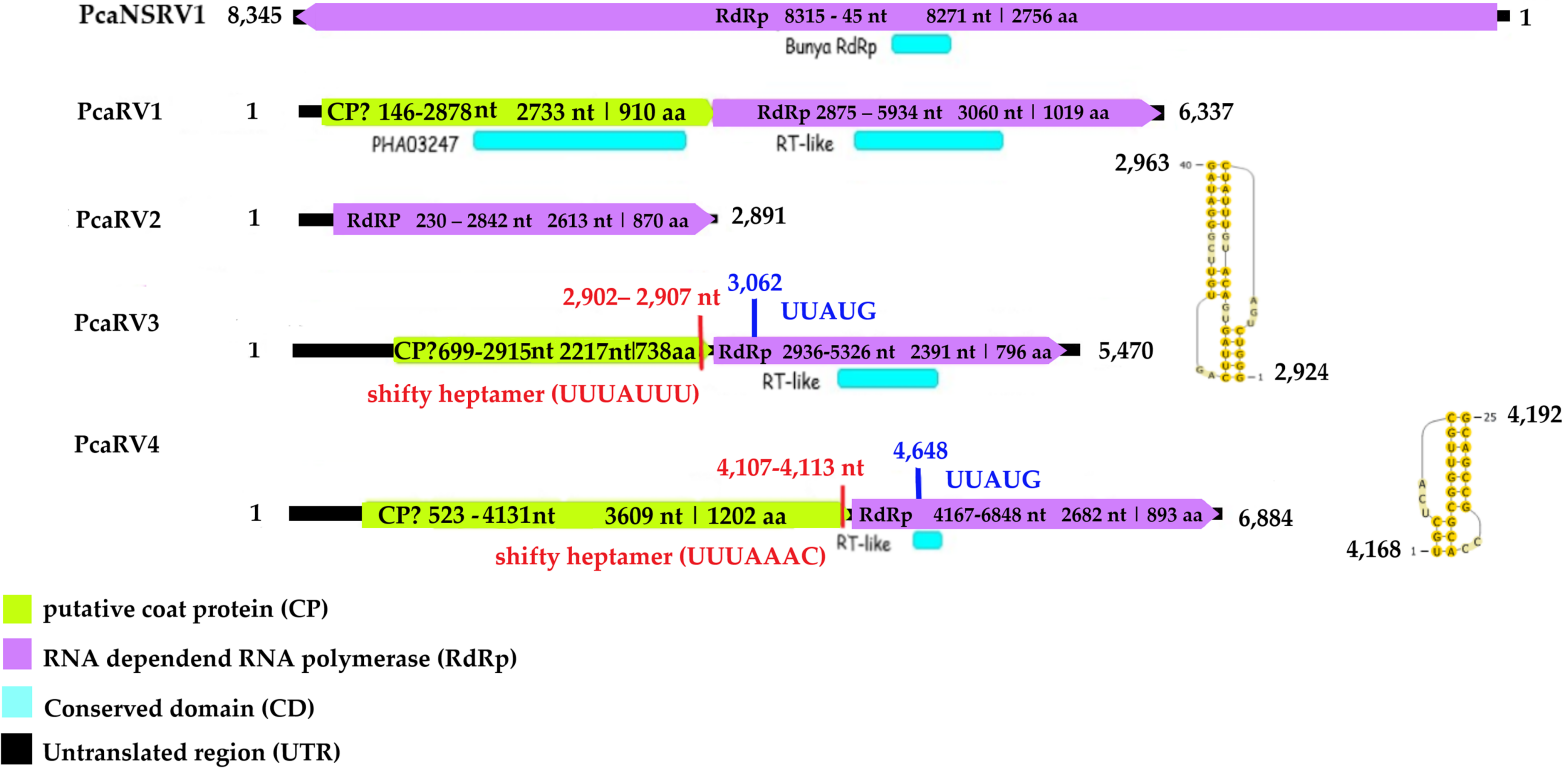
Genome organization of viruses PcaRV1-4 and PcaNSRV1. Genome sequence of PcaRV5 is not graphically represented as it is partial and requires further characterization.

**Figure 3:**
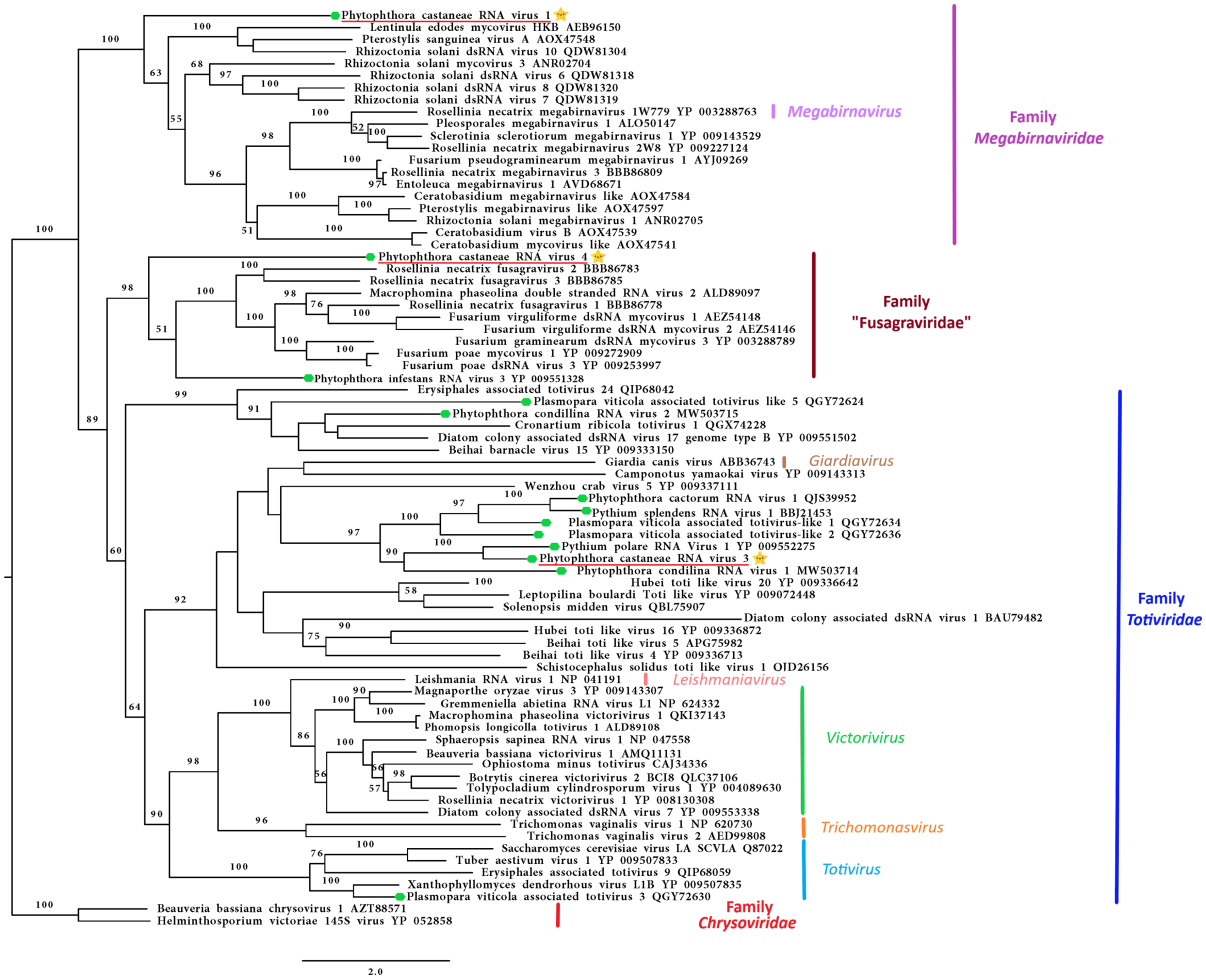
Phylogenetic RAxML tree including the three dsRNA viruses described in this study, PcaRV1 (megabirna-like virus), PcaRV3 (toti-like virus), and PcaRV4 (fusagra-like virus; all three indicated by a yellow star 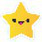), and related members belonging to the families *Megabirnaviridae*, *Toriviridae* proposed family “Fusagraviridae.” The viruses described from oomycetes are indicated by a green hexagone 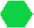. Nodes are labeled with bootstrap percentages ≥50% only. Family classification and the corresponding pBLAST accession numbers are shown next to the virus names. The tree is rooted in the midpoint. Branch lengths are scaled to the expected underlying number of amino acid substitutions per site. The scale bar is indicatig 2.0 aa substitutions per site, per branch.

Following the pipeline in which host reads were removed prior to *de novo* assembly, one single contig of 6,006 nts was detected. Following the other pipeline where the host reads were not removed, a slightly bigger contig of 6,337 nts was retrieved from the analyzed data. The two putative virus sequences are identical in ORFs regions. The two contigs differ in four nts in the 3’ UTR region. The sequence of 6,337 nts was chosen as a representative sequence for Phytophthora castaneae RNA virus 1 (PcaRV1). The putative virus shows similarities to the large (L) segment of megabirnaviruses and has a 60.2% GC content, two overlapping ORFs likely coding for the CP (ORF 1–5′ proximal) and the RdRp (ORF2-3′ proximal; Figure 2). The overlapping region consists of 4 nts (AUGA) including the stop codon of the ORF 1 and the start codon of the ORF2. Interestingly, two continuous methionine codons occur at the beginning of the ORF2. No typical heptanucleotide slippery site or shifty heptamer motif facilitating the −1 programmed ribosomal frameshifting was observed at the end of the ORF1 with the general sequence X XXY YYZ (spaced triplets represent pre-frameshift codons; Bekaert et al., 2005) where X represents A/G/C/U, Y defines A/U, and Z represents A/C/U. Likewise, neither a possible slippery site similar to those found in Sclerotinia sclerotiorum megabirnavirus 1 (SsMBV1; AAAAAAC; Wang et al., 2015) or Fusarium pseudograminearum megabirnavirus 1 (FpgMB1; GAAAAAC; Zhang et al., 2018) was detected. Also, no clear H-type pseudoknot was found upstream of the ORF2. According to the BlastX search, PcaRV1 shares the highest identity with Charybdis toti-like virus (Table 1), a divergent virus with uncertain placement inside *Ghabrivirales* and undetermined genome organization due to lack of a full sequence being available (Charon et al., 2021). PcaRV1 shows sequence homology also to the FpgMB1 (query cover: 28%; E value:1e^−51^; identity; and 31.30% accession: AYJ09269.1). The putative dsRNA1 of PcaRV1 shows a 29.15% identity (query cover: 25%; E value: 9 e^−50^; and accession: QTF98696.1) to the Rosellinia necatrix megabirnavirus 2 (RnMBV1). With Rhizoctonia solani RNA virus HN008 (RsRV-HN008), PcaRV1 shares 26.60% identity in its RdRp region (query cover: 22%; E value: 6 e^−27^; and accession: YP_009158860.1). The second segment of PcaRV1 was not detected based on the initial BlastX search against the virus protein database. Thus, the dsRNA2 was searched among Trinity contigs encoding for proteins having no detectable homology with proteins of the NCBI nr database (e.g., ORFan contigs; Supplementary Table 10), but no reliable dsRNA2 candidate could be retrieved. The CDD search detected two conserved domains in both ORFs (Figure 2). In the ORF2, a RdRp domain RT_like family (E value 6.08 e^−20^; interval 4,215–5,243 nts) was detected. Besides, in the interval from 1,547 to 3,028 nts of the ORF1 with unknown function, the search revealed a significant record (E value 1.09 e^−04^) with the provisional large tegument protein UL36 (PHA03247; PHA03247), which is the largest protein of herpesviruses. Multiple alignment of the RdRp aa sequence of the PcaRV1 with those of other related viruses enabled detection of conserved sequence motifs (I-VIII; Supplementary Figure 3). To determine the phylogenetic position of PcaRV1, the RdRp protein sequence of this virus was compared with RdRp sequences of other related unclassified viruses and viruses of families *Totiviridae*, *Megabirnaviridae,* and proposed family “Fusagraviridae.” Phylogenetically, PcaRV1 is related to unclassified megabirnaviruses and, together with them, putatively falls into the *Megabirnaviridae* virus family (Figure 3). Megabirnaviruses are found to infect filamentous fungi (Sato et al., 2019). PcaRV1 would be the first megabirnavirus discovered in oomycetes and the genus *Phytophthora*, respectively.

#### Narna-Like Virus

**Figure 4:**
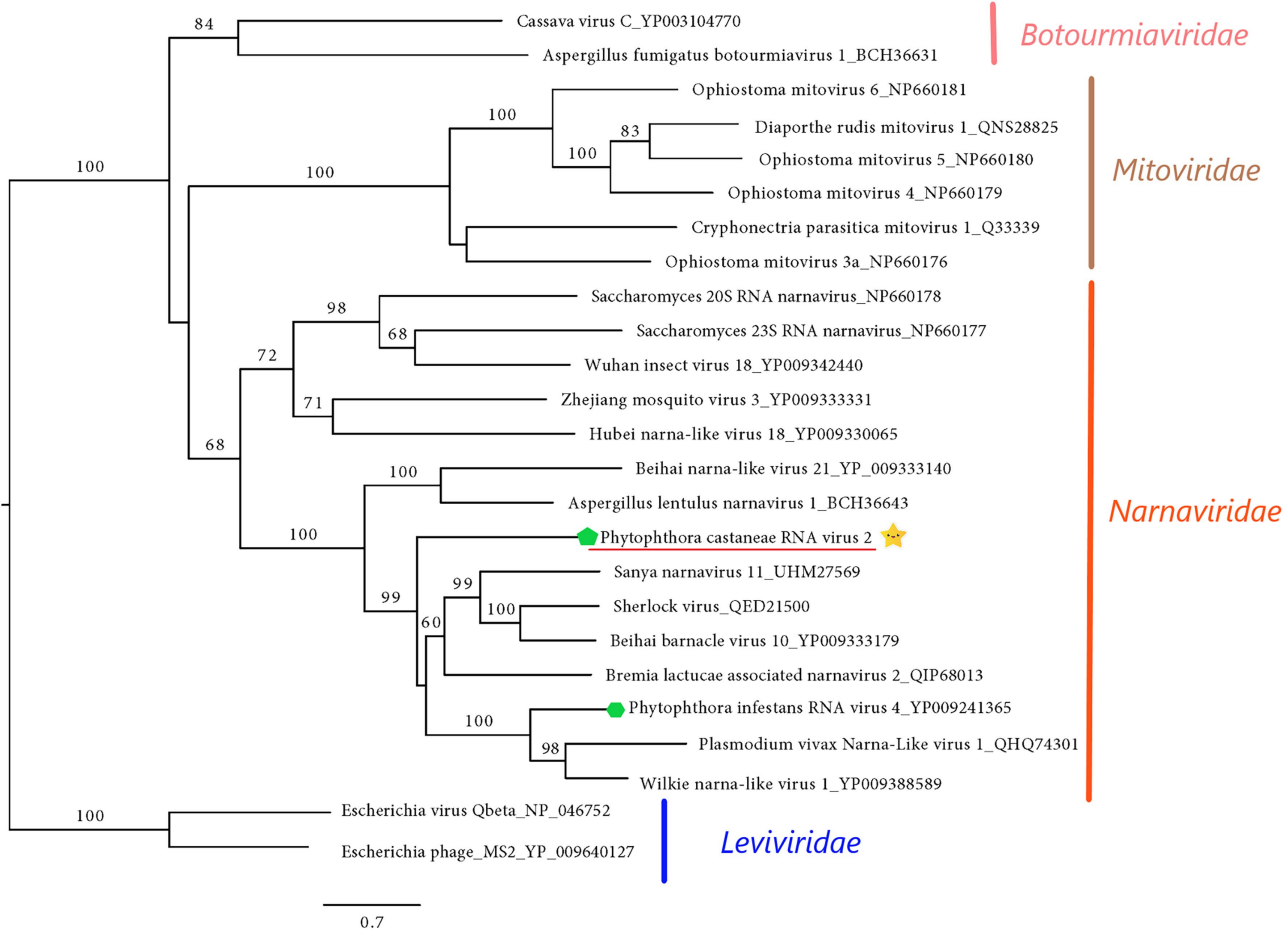
Maximum likelihood tree (RAxML) showing the phylogenetic relationships of PcaRV2 (indicated by the yellow star 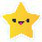) with other (+)ssRNA viruses belonging to families *Narnaviridae*, *Mitoviridae*, *Leiviviridae*, and *Botourmiaviridae*. The viruses described from oomycetes are indicated by a green hexagone 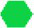. Nodes are labeled with bootstrap percentages ≥50%. Branch lengths are scaled to the expected underlying number of amino acid substitutions per site. Family classification and the corresponding pBLAST accession numbers are shown next to the virus names. The tree is rooted in the family *Leviviridae*.

The final sequence of Phytophthora castaneae RNA virus 2 (PcaRV2) is 2,891 nts long, coding a single protein, putatively RdRp (Figure 2). The sequence has a GC content of 49.2% and one single ORF putatively corresponding to the RdRp. The ORF size is 2,613 nts or 870 aa. No conserved domains were detected within the PcaRV2 genome sequence (Figure 2). However, when the aa sequence of the ORF1 was aligned to RdRp of other narnaviruses, conserved motifs from III to VII were detected (Supplementary Figure 4). A phylogenetic tree was constructed using (aa) sequences of the RdRp of PcaRV2 together with 24 other (+)ssRNA viruses grouped under the families *Narnaviridae*, *Mitoviridae*, *Botourmiaviridae,* and *Leviviridae* (Figure 4). The ML tree shows that PcaRV2 clusters with narnaviruses and is phylogenetically close to Phytophthora infestans RNA virus 4 (Figure 4). According to the BlastX results, the most similar virus to PcaRV2 is the Sanya narnavirus 11 (UHM27569.1; Table 1).

#### Toti-Like Virus

The final contig corresponding to the genome of Phytophthora castaneae RNA virus 3 (PcaRV3) has a length of 5,470 nts and 59.3% GC content. It has two overlapping ORFs, ORF1 (3’end-proximal) putatively encoding the CP and ORF2 (5′ end-proximal) corresponding to the RdRp. Conserved motifs of the RdRp (I-VIII) have been detected by the CDD search (Figure 2; Supplementary Figure 3). The ORFs overlap by 70 nts (nucleotide (nt) positions 2,846–2,915), which include the stop codon for ORF 1 (UAG) and the start codon for ORF2 (GUG, Valine). GUG is an alternative non-canonical codon that, under certain circumstances, can also be recognized as an initiator codon in RNA viruses. A heptanucleotide slippery site or shifty heptamer motif was observed at the end of the ORF1 (UUUAUUU, nt positions 2,902– 2,907), which may facilitate −1 programmed ribosomal frameshifting in PcaRV3 transcripts. A short spacer region of 11 nts appears to precede an H-type pseudoknot between nt 2,924 and 2,963 (Figure 2) from the slippery site. Upstream at nt position 3,062 UUAUG is found, a key motif for translation of the downstream ORF. PcaRV3 genome organization and its phylogenetic grouping (Figure 3) indicate that PcaRV3 belongs to *Totiviridae*, a family of linear uncapped dsRNA viruses approximately 4.6 to 7.0 kb in size (Wickner et al., 2012). According to Blast analyses, PcaRV3 is most similar in its RdRp region to Totiviridae sp. (UHS72506.1; Table 1) but it also shares 49.34% of identity with RdRp of Pythium polare RNA virus 1 (YP_009552275.1; Sasai et al., 2018), 33.62% with Plasmopara viticola lesion associated totivirus-like 1 (QGY72634.1; Chiapello et al., 2020), 35.01% with Phytophthora condilina RNA virus 1 (QTT60989.1; Botella and Jung, 2021), and 32.86% with Phytophthora cactorum RNA virus 1 (QUE45741.1; Poimala et al., 2021) and to other toti-like viruses (data not shown). The phylogenetic tree shows that PcaRV3 clusters with Phytophthora condilina RNA virus 1 and Pythium polare RNA virus 1 (Figure 3).

#### Fusagra-Like Virus

The final sequence of a virus designated Phytophthora castaneae RNA virus 4 (PcaRV4) is 6,884 nts long. PcaRV4 has two large discontinuous ORFs (Figure 2), ORF1 (putative CP, nt 517–4,131) and ORF2 (RdRp, nt 4,167–6,848). The elements needed to accomplish −1 ribosomal frameshifting in some RNA viruses were found. Shifty heptamer (UUUAAAC) at nts 4,107 to 4,113; a spacer region (54 nts from the slippery site) the recording stimulatory element consisting of an H-type pseudoknot structure between nts 4,168 and 4,192, and finally, 534 nts downstream at nt 4,648, the UUAUG motif. A putative conserved domain containing RNA-dependent RNA polymerase superfamily 4 (pfam02123: RdRP_4) was detected by CDD search in translated ORF2 at the position from 4611 to 5822 nts (E value 8.37e^−30^; Figure 2; Supplementary Figure 3). Pfam02123 family includes RdRp proteins of members of the genera *Luteovirus*, *Totivirus,* and *Rotavirus* and it is a member of the superfamily cl02808. According to the BlastX search, PcaRV4 is associated with proteins of viruses putatively belonging to the newly proposed family “Fusagraviridae.” The ORF1 of PcaRV4 is most similar to Bremia lactucae associated fusagravirus 1 ORF2 (QIP68010.1) with 28.15% identity and 59% coverage (E value 1e^−47^), hypothetical protein 1 of Wuhan insect virus 28 (YP_009342429.1) with which it shares 22.48% similarity (query coverage 41%; E value 5e^−18^) and to hypothetical protein EXH54_gp1 of Phytophthora infestans RNA virus 3 (YP_009551327.1), with 29.96% but less coverage (23%). ORF1 is also similar to Phytophthora infestans RNA virus 3 (YP_009551328.1), RdRp of Bremia lactucae associated fusagravirus 2 (QIP68015.1), hypothetical protein of Macrophomina phaseolina fusagravirus 1 (QDM35289.1), and other viruses. In its RdRp region, PcaRV4 is most similar to RdRp of Bremia lactucae associated fusagravirus 1 (QIP68009.1), with which it shares 29.96% (query coverage 59%; E value 1e^−45^) but also with RdRp of Phytophthora infestans RNA virus 3 (YP_009551328.1), RdRp of Spissistilus festinus virus 1 (YP_003800001.1), and other viruses. The whole sequence is most similar to ORF2 of Bremia lactucae associated fusagravirus 1 (QIP68010.1; Table 1). The phylogenetic tree including viruses of the families *Megabirnaviridae*, *Totiviridae,* and putative members of proposed “Fusagraviridae” (Figure 3) revealed that PcaRV4 clusters with the fusagraviruses but groups separately with PiRV3, indicating a close phylogenetic relationship with this virus, also described from *Phytophthora*. When short sRNA reads of the four libraries were mapped individually to the PcaRV4 genome, high coverage was achieved. However, due to its low similarity with known viruses, the BlastX search failed in identification of the short contigs representing PcaRV4 produced by Velvet in isolates VN999, VN1008, and VN1012, respectively. By BlastX, such contigs were identified only in isolate VN1004, although the genomic sequence of PcaRV4 was obtained from Trinity assemblies of isolate VN999 and almost its entire length was confirmed by RT-PCR and Sanger sequencing in all four Vietnamese isolates.

#### Bunya-Like Virus

**Figure 5:**
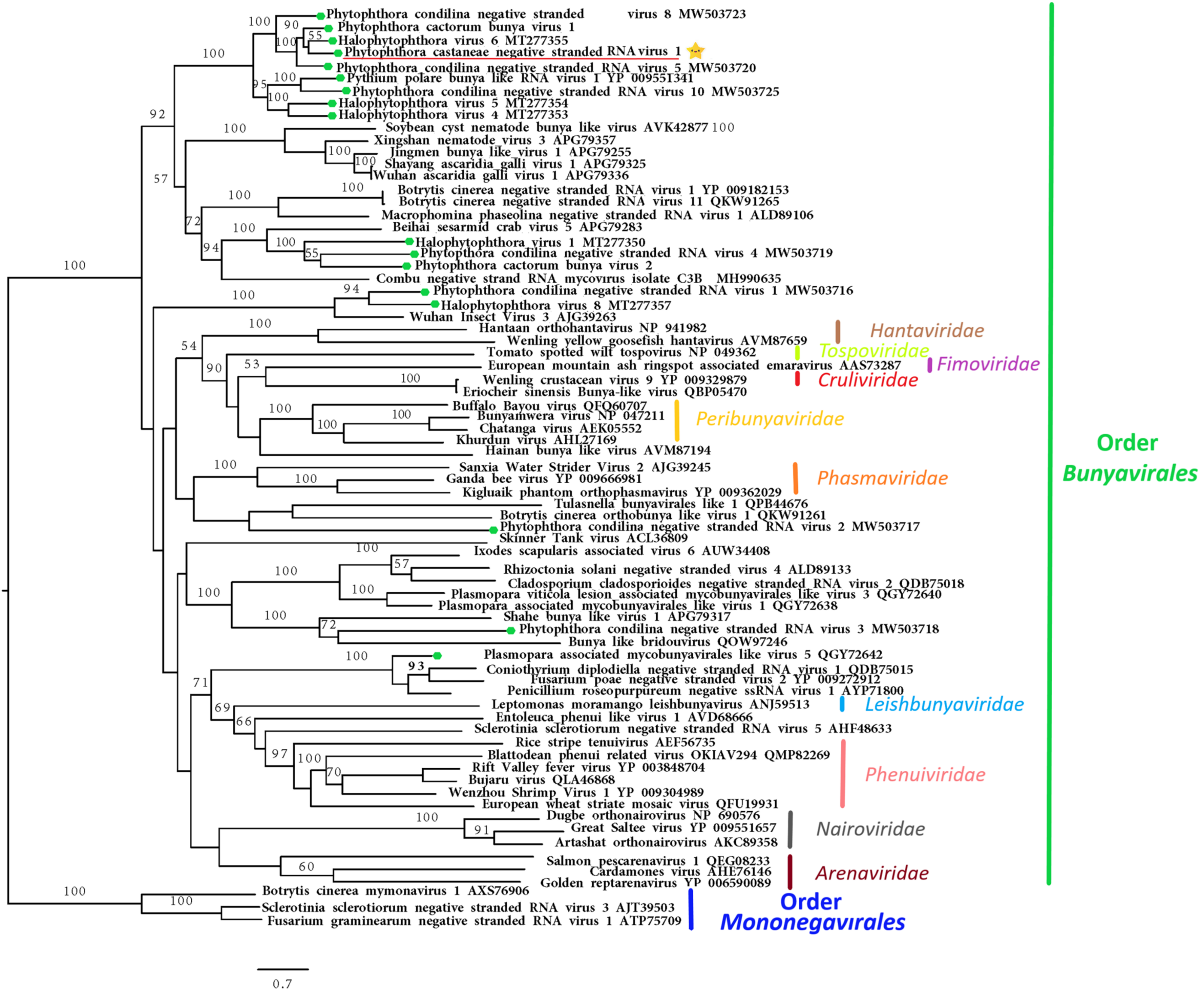
Maximum likelihood tree (RAxML) depicting the phylogenetic relationship of the predicted RdRp of PcaNSRV1 (indicated by the yellow star 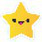) with other complete RdRp belonging to related (−)ssRNA viruses from the orders *Bunyavirales* and *Mononegavirales*. The viruses described from oomycetes are indicated by a green hexagone 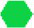. Nodes are labeled with bootstrap support values. Branch lengths present calculated evolutionary distannce and are scaled to the expected underlying number of amino acid substitutions per site. Nodes are marked with bootstrap percentages ≥50% only. The tree is rooted in *Mononegaviales* viruses, which are classified within the family *Mymonaviridae*. Family classification and the corresponding pBLAST accession numbers are shown next to the virus names. The scale bar is indicatig 0.7 aa substitutions per site, per branch.

The nearly complete (−)ssRNA genome of a virus designated Phytophthora castaneae negative-stranded RNA virus 1 (PcaNSRV1) was 8,345 nts long, with one single 8,271 nts long ORF (nt 8,315 to 45), putatively coding for a polyprotein/RdRp (Figure 2) and L segment of viruses in the order *Bunyavirales*. The phylogenetic analysis based on the partial RdRp (aa) sequence of PcaNSRV1 and those of related viruses revealed that PcaNSRV1 clusters with unclassified bunyaviruses of other *Phytophthora* species, i.e., *P. cactorum* (Poimala et al., 2021), *P. condilina* (Botella and Jung, 2021), and those described from the genus *Halophytophthora* (Botella et al., 2020; Figure 5). According to the BlastX search, PcaNSRV1 is most similar to Phytophthora cactorum bunyavirus 1, with whom it shares 57.16% identity (Table 1). PcaNSRV1 is also similar to Phytophthora condilina negative-stranded RNA virus 5 (PcoNSRV5; query coverage 99%; E value 0.0; and percent identity 52.14%), Halophytophthora RNA virus 6 (HRV6; query coverage 68%; E value 0.0; and percent identity 62.47%), Phytophthora cactorum bunyavirus 2 (PcBV2; query coverage 96%; E value 0.0; and percent identity 46.83%), and others. Conserved motifs of bunya RdRp (pfam04196), the only member of superfamily cl20265, were detected in the region from 945 to 1415 aa (E value 6.16e^−07^; Figure 2; Supplementary Figure 5). In addition, pfam15518: L_protein_N endonuclease domain was detected at aa position 245–298 (E value 4.18e^- 03^). Many bunyavirus L proteins have this domain at the N-terminus (Reguera et al., 2010).

#### Endorna-Like Virus

As the RNA of isolate VN1008 was sequenced only by HTS of sRNAs, and contigs of viruses similar to *Endornaviridae* were not detected in VN999 *de novo* assembled total RNA reads, contigs 139, 274, and 295 (Supplementary Table 6) were used for primer design and Sanger sequencing of 2,182 nts long sequence resembling members of *Endornaviridae*. Virus-specific primers (Supplementary Table 2) were designed to fill sequence gaps between the short contigs, and the amplicons obtained through RT-PCR were Sanger sequenced in both directions allowing sequence identification of a longer sequence of a virus here Phytophthora castaneae RNA virus 5 (PcaRV5). PcaRV5 shares the most similarity with Diatom colony associated dsRNA virus 15 (DcaRV15; Table 1). It is 33.42% identical to the polyprotein of Erysiphe cichoracearum alphaendornavirus (YP_009225663.1; query coverage 99%; E value 6e^−119^), 48.92% to the polyprotein of Chalara endornavirus CeEV1 (ADN43901.1; query coverage 57%; E value 1e-^118^), and 46.14% to the polyprotein of Phytophthora cactorum alphaendornavirus 3 (QUA12642.1; query coverage 67%; E value 5e^−117^). Conserved domain of pfam00978 RdRP_2 super family (E value 2.88e^−08^; accession cl03049) was detected at interval from 1,544 to 2,014 bp of nucleotide sequence. A longer sequence of PcaRV5 was not determined and the putative virus was not further characterized.

### Intraspecific Genetic Variability of *Phytophthora castaneae* Viruses

The intraspecific genetic variability of PcaRV1-4 and PcaNSRV1 hosted by the four *P. castaneae* isolates was examined using Sanger sequencing. The intraspecific genetic variability among virus strains present in the four isolates of *P. castaneae* appeared to be very low and homogeneous. Thus, the overall pairwise (pw) sequence comparison (PASC) percentage between PcaRV1 nucleotide sequences (strains) was 99.8% for an alignment of 5,985 bp; in PcaRV2 strains alignment (length: 1,625 bp), it was 98.8%; for PcaRV3 strains, it was 98.9% (alignment length: 4,306 bp); for PcaRV4, it was 98.5% (alignment length: 1,895 pb); and for PcaNSRV1 strains, PASC had the lowest value, 95.6% (alignment length: 7,858 bp). Within PcaNSRV1, the highest pw variability was shown between the strain VN1008 and the rest of the strains (PASC 91.21–91.23%), while the pw similarity between VN999, VN1004, and VN1012 ranged 99.89–99.97%.

### Depth of Coverage

The high number of reads and depth of coverage show that the five virus genomic sequences obtained from Trinity assembly are properly supported. PcaRV1 had the highest number of reads mapped to its almost complete genome in all four sRNA datasets, as well as the deepest coverage in all sRNA libraries, but one (Table 2). Overall, PcaNSRV1 is the virus with the lowest coverage depth, although it did not have the lowest number of reads in all the four data sets. In the library of isolate VN999, PcaRV3 showed the smallest number of reads mapped to its genome in comparison to other viruses (Table 2). According to total RNA seq, the virus with the most reads covering its genome was PcaRV1 that also had the deepest coverage, while PcaRV4 was covered the least (Table 2).

**Table 2:** Information about the depth of coverage and the total number of sRNA and total RNA reads mapped to each virus.

Virus^I^	Read length total RNA seq VN999	Reads in total RNA seq VN999^II^	Depth of Coverage total RNA seq (VN999)^x^	Read length sRNA seq (VN999 VN1008)^I,II^	Read length sRNA seq (VN1004 VN1012)^I,II^	Reads sRNA seq (VN999)^II^	Depth of coverage in VN999^*^	Reads sRNA seq (VN1004)^II^	Depth of coverage in VN1004^*^	Reads sRNA seq (VN1008)^II^	Depth of coverage in VN1008^*^	Reads sRNA seq (VN1012)^II^	Depth of coverage in VN1012^*^
PcaRV1	101	2,427,087	38,683	36	50	217,861	1,238	226,485	1,787	168,840	959	295,192	2,329
PcaRV2	101	250,248	8,743	36	50	58,916	734	68,560	1,186	121,361	1,511	102,384	1,771
PcaRV3	101	777,368	14,354	36	50	18,935	125	59,923	548	13,679	90	49,552	453
PcaRV4	101	60,512	889	36	50	72,976	382	136,977	995	73,740	386	183,367	1,332
PcaNSRV1	101	285,239	3,453	36	50	24,637	106	21,332	128	6,624	29	25,261	151

I Virus, Acronym of a virus.

II Reads sRNA seq^*^, the number of reads that mapped to each virus.

III Read length (insert size) of sRNA and total RNA libraries.

x The depth of coverage was calculated using a formula specified in the Materials and Methods section.

## Discussion

A three-method approach including two HTS methods, small and stranded total RNA sequencing, and the conventional dsRNA extraction method was employed in order to characterize and have a better understanding of the viral community hosted by the oomycete pathogen *P. castaneae*.

In the current study, the dsRNA detection technique provided first insights into the potential occurrence of viruses and, based on the presence of dsRNA banding patterns, isolates were chosen for further sequencing analyses. Linking sizes of dsRNA molecules with the described viruses was attempted, but the patterns were not consistent through all performed extractions (Supplementary Figure 1). Furthermore, due to the similar virus genome sizes, i.e., PcaRV1 and PcaRV4 larger segments of 6,337 bp and 6,884 bp, respectively, it would have been difficult to confirm the corresponding dsRNA bands of each virus.

Concerning the two tested HTS approaches, RNA-Seq was found superior to sRNA sequencing for virus detection and identification in *P. castaneae*. While sRNA sequencing was found not to be the best method for that purpose, this study demonstrates that *P. castaneae* hosts a diverse virome processed into sRNAs, suggesting that *P. castaneae* has an active RNAi mechanism. To our knowledge, *P. castaneae* is the first *Phytophthora* species reported with such a diverse virome and high number of viruses from different viral families co-occurring in a single isolate, although an abundant occurrence of viral co-infections and extreme virus prevalence among host isolates was also found in *P. cactorum* (Poimala et al., 2021). Three of the analyzed *P. castaneae* isolates (VN999, VN1004, and VN012) appear to host viruses belonging to the families *Megabirnaviridae*, *Narnaviridae*, *Totiviridae*, “Fusagraviridae,” and the order *Bunyavirales*. However, in isolate VN1008, in addition to the above-mentioned viruses, the occurrence of a putative endornavirus was also detected by sRNA sequencing. It is common for mycoviruses to co-infect and interact in a variety of ways, forming interactions from synergistic to neutral and antagonistic (Thapa and Roossinck, 2019). Multiple virus infections from more than one virus family have been previously reported in *P. infestans* (hosting a narnavirus and a virus related to the proposed family “Fusagraviridae”; Cai et al., 2012, 2013), *P. condilina* (toti-like and bunya-like viruses; Botella and Jung, 2021), and *P. cactorum* (bunya-like, toti-like, alpahendronaviruses, and an usti-like virus; Poimala et al., 2021). Likewise, multiple virus infections seem to be common in other oomycete species including *Py. polare* hosting dsRNA (toti) and (−)ssRNA (bunya) viruses (Sasai et al., 2018), and *Pl. viticola*-associated lessions in which 283 novel viruses resembling genomes of (+)ssRNA, (−) ssRNA, and dsRNA from different virus families and ORFan RdRp viral segments were identified using a metagenomics approach. Interestingly, members of the families *Narnaviridae* [(+)ssRNA], *Totiviridae* (dsRNA), and the order *Bunyavirales* [(−)ssRNA] have been described in the majority of oomycete species screened for viruses so far, suggesting a strong coevolution history of these virus clades with oomycetes. Multiple viral infections are also reported from various fungal plant pathogens (Doherty et al., 2007; Deakin et al., 2017; Botella and Hantula, 2018; Hillman et al., 2018; Zhu et al., 2018; Hantula et al., 2020) but, as indicated above, they seem to be particularly prevalent in oomycetes. As oomycete hyphae lack septa, it has been speculated that viruses are able to passively move with the flowing cytoplasm through the mycelium (Poimala et al., 2021).

This is the first study to report a megabirna-like virus on the genus *Phytophthora*, or any other oomycete genus. PcaRV1 appears to be the first megabirna-like virus detected outside of the kingdom of Fungi. *Megabirnaviridae* is a family of viruses with non-enveloped linear dsRNA genomes organized in two segments, dsRNA 1 (8.9 kb) and dsRNA 2 (7.2 kb; in total 16.1 kb), each with two ORFs. The only known member of the family *Megabirnaviridae* and its single genus *Megabirnavirus* is Rosellinia necatrix megabirnavirus 1 (RnMBV1; Chiba et al., 2009; Sato et al., 2019). Since the genus is monospecific, species demarcation criteria are not yet defined, and no other viruses have been assigned to the family or the genus (Sato et al., 2019). Despite the description of many viruses in other filamentous fungi that share similar characteristics and phylogenetic positions to RnMBV1 such as SsMBV1 (Wang et al., 2015), Rosellinia necatrix megabirnavirus 2 (Sasaki et al., 2016), Pleosporales megabirnavirus 1 (Nerva et al., 2016), Entoleuca megabirnavirus 1 (Velasco et al., 2018), Rosellinia necatrix megabirnavirus 3 (Arjona-Lopez et al., 2018), Rhizoctonia solani megabirnavirus 1 (Bartholomäus et al., 2016), RsRV-HN008 (Zhong et al., 2015), and FpgMB1, these viruses remain unclassified (Sato et al., 2019). The genomic properties of PcaRV1 are similar to other reported megabirnaviruses except for the seemingly smaller size (*ca.* 6.4 kb) of its larger segment (dsRNA1). However, as the 3′- and 5′- termini were not confirmed, we cannot rule out the possibility of the larger segment being longer. The putative smaller segment (dsRNA 2) of PcaRV1 was not detected in the total RNA seq data. Similarly, the second segment has not been reported from some other megabirnaviruses. For example, RsRV-HN008, characterized from *Rhizoctonia solani* J.G. Kühn, has a genome of 7,596 nts organized on one segment containing two non-overlapping coding regions (Zhong et al., 2015). Detection of PcaRV1 by sRNA seq suggests it is targeted by the oomycete RNAi system.

Phylogenetic analyses of this study, based on the partial RdRp sequences, placed virus PcaRV3 from *P. castaneae*, together with putative members of the virus family *Totiviridae*. PcaRV3 also has a genome organization and size typical for a toti-like virus. According to the International Committee on Taxonomy of Viruses (ICTV) 2021 report,^6^ the family is organized into five genera, i.e., *Giardiavirus*, *Leishmaniavirus,* and *Trichomonasvirus,* all associated with infections of protozoa, and *Totivirus* and *Victorivirus* infecting fungi. However, many viruses with similarities to totiviruses are unclassified and the host range is much wider than previously assumed (De Lima et al., 2019). Totiviruses are correlated with latent infections in fungal and protozoan hosts (Wickner et al., 2012), but they have also been reported from fish, plants, insects, and oomycetes (Koyama et al., 2015; Chen et al., 2016; Martinez et al., 2016; Mor and Phelps, 2016; Sasai et al., 2018; De Lima et al., 2019; Poimala and Vainio, 2020; Botella and Jung, 2021; Poimala et al., 2021). *Totiviridae* members have distant phylogenetic relationships with the megabirnaviruses, and both belong to the order *Ghabrivirales* (Sato et al., 2019). Similarly, to the dsRNA1 of megabirnaviruses, the RdRP gene of totiviruses of the genera *Totivirus* and *Victorivirus* is encoded downstream of the CP gene. The *Totiviridae* whose dsRNA genomes are not divided are phylogenetically distinct from megabirnaviruses. Coding strands of these totiviruses have shorter UTR at 5′ (<0.5 kb) and smaller virus particles of *ca.* 40 nm diameter (Sato et al., 2019).

The putative new fusagravirus, PcaRV4, has a relatively long 5′ UTR of 522 bp and a small 36 nts long 3′ end UTR. The 5’ UTR of fusagraviruses ranges in size from 865 to 1,310 bp, while 3′ UTR can be from 7 to 131 bp long. PcaRV4 is slightly smaller (6,884 bp) than other viruses putatively grouped in the proposed “Fusagraviridae” family, but the full length of its genome is not confirmed. Genome organization and phylogenetic placement of PcaRV4, as well as the occurrence of programmed −1 ribosomal frameshifting and presence of putative shifty heptamer motif located immediately upstream of the stop codon of ORF1, are in accordance with other “Fusagraviridae” members (Wang et al., 2016). PcaRV4 was also detected by sRNA sequencing, suggesting that its presence in the cell activates the RNAi mechanism of *P. castaneae*.

*Bunyavirales* is an order of (−)ssRNA viruses accommodating 12 families infecting a broad range of hosts including plants, vertebrates, and invertebrates, and includes the genus *Coguvirus* unassigned to any viral family with a single species Citrus coguvirus associated with citrus disease (Abudurexiti et al., 2019). The number of genomic segments of bunyaviruses can vary from two to six. However, most commonly, their genomes are organized in three ss segments, designated according to their size: the small (S; *ca.* 1–2 kb), medium (M; *ca.* 3.7–5 kb), and the large (L) segment (*ca.* 6.8–12 kb; Garrison et al., 2020; Hughes et al., 2020; Leventhal et al., 2021). The L segment codes for a single protein, the RdRp. The M segment codes for Gn and Gc glycoproteins and sometimes a non-structural protein (NSm). The S segment codes for nucleocapsid protein (N) and, typically, a non-structural protein (NSs). The RNA genomes of these viruses are coated with N protein which, together with L protein, form ribonucleoprotein complexes (RNPs; Reguera et al., 2010). PcaNSRV1 codes for a single protein, the RdRp, representing the L segment. No other segments of this virus, except the L segment, were retrieved from either total RNA or sRNA sequencing data after BlastX and BlastN searches. The absence of the putative NP and other non-structural (Ns) associated proteins may indicate their low copy numbers or low level of conservation, making it difficult to detect them through a homology search (Botella and Jung, 2021). The size and genome organization of PcaNSRV1 are in accordance with other *Bunyavirales* members and bunya-like viruses found in *P. cactorum* (Poimala et al., 2021), *P. condilina* (Botella and Jung, 2021), and *Halophytophthora* sp. (Botella et al., 2020), in which no segments other than L were identified.

According to the ICTV (see footnote 7), the family *Narnaviridae* has only one genus *Narnavirus*, containing only two species, *Saccharomyces 20S narnavirus* and *Saccharomyces 23S narnavirus* described from the yeast *Saccharomyces cerevisiae* Meyen ex E.C. Hansen. Besides these two officially recognized viral species, a number of narna-like viruses have been described in a range of other organisms including arthropods (Shi et al., 2016; Harvey et al., 2019), algae (Waldron et al., 2018), fungi (Osaki et al., 2016), oomycetes (Cai et al., 2012), mosquitoes (Cook et al., 2013; Chandler et al., 2015; Shi et al., 2017; Göertz et al., 2019), protozoa (Akopyants et al., 2016; Lye et al., 2016; Grybchuk et al., 2018), and in association with *Plasmodium* parasites causing human malaria (Charon et al., 2019). Interestingly, apart from typical narna- and narna-like viruses with genetic information organized on one segment, several other narna-like viruses with bi- and polysegmented genomes have been described in recent years. The first observed bisegmentation of the narna-like virus genome was reported in Leptomonas seymouri Narna-like virus 1 (Grybchuk et al., 2018) and in Matryoshka RNA virus 1 (Charon et al., 2019). In addition, narna-like viruses with a split polymerase palm domain “splipalmiviruses” have been discovered in the ascomycetus fungus *Oidiodendron maius* Barron (Sutela et al., 2020) and similar multisegmented narna-like viruses with a divided RdRp gene were reported from other ascomycetes, i.e., *Aspergillus fumigatus* Fresen., *Magnaporthe oryzae* B.C. Couch (Chiba et al., 2020), *Sclerotinia sclerotiorum* (Lib.) de Bary (Jia et al., 2021), and *Botrytis cinerea* Pers. (Ruiz-Padilla et al., 2021). “Orfanplasmoviruses” have been discovered in the virome associated with the downy mildew *Pl. viticola* (Chiapello et al., 2020) and in the conifer fungal pathogen *Heterobasidion parviporum* (Sutela et al., 2021). In some narnaviruses, negative-sense coding ORFs were observed (rORFs; DeRisi et al., 2019; Dinan et al., 2020). Due to recent discoveries of a number of remarkable features of narna-like viruses, several studies suggest the establishment of novel families, genera, and clades in order to accommodate the diverse groups of narnaviruses (Chiapello et al., 2020; Dinan et al., 2020; Ruiz-Padilla et al., 2021). The virus PcaRV2 has a size, genome organization, and phylogenic position in accordance with traditional, monosegmented members of *Narnaviridae*, indicating that it indeed represents a putative novel member of this family.

The family *Endornaviridae* contains linear (+)ssRNA viruses with genome sizes ranging from 9.7 to 17.6 kb. There are two genera within this family, to which endornaviruses are assigned based on the size of their genome, presence of distinct domains, and their host association (Valverde et al., 2019). *Alphaendornavirus* genera members are found infecting plants, oomycetes (Fukuhara and Gibbs, 2012), and fungi. *Betaendornavirus* includes viruses infecting ascomycete fungi (Valverde et al., 2019). Based on the low similarity of the *ca.* 2 kb fragment of PcaRV5 to other previously described endornaviruses, we conclude that this virus may represent a putative novel species belonging to the genus *Alphaendornavirus*. However, the full-length genome of this virus needs to be characterized to allow correct determination of its taxonomical position. As aforementioned alphaendornaviruses appear to be common in *Phytophthora* species. Interestingly, two alphaendornaviruses reported from Japanese isolates pathogenic to asparagus, Phytophthora endornavirus 2 (PEV2) and Phytophthora endornavirus 3 (PEV3) were found to inhibit hyphal growth and stimulate production of zoosporangia. In addition, PEV2 and PEV3 reportedly had a possible effect on both reduced as well as increased fungicide sensitivity of their host isolates (Uchida et al., 2021). Effects of viruses on their oomycete host have been also reported from the potato-late blight pathogen *P. infestans* infected by PiRV-2. PiRV-2 has been demonstrated to stimulate sporangia production in its host isolates, having a possible impact on ecological fitness of *P. infestans*, probably *via* downregulation of ammonium and amino acid intake (Cai et al., 2019b).

*P. castaneae* isolates VN999, VN1004, VN1008, and VN1012 are part of an ongoing broader study on viral diversity of *Phytophthora* Clade 5 and exploration of the possible effect of these viruses on their hosts. Repetitive attempts using thermo- and chemotherapy have been made in order to cure the isolates of viruses and study the isogenic strains for phenotypic effects (M. Raco unpublished data). However, due to the high viral load, no virus-free isolates have been obtained so far. Such rich mixed viral infections as observed in the studied isolates of these *P. castaneae* might have an effect on the overall energy consumption and vitality of the oomycete, which could be related to our observation that compared to other isolates these *P. castaneae* isolates have reduced survival time in storage and need more frequent transfers when compared to other isolates of PRC collection. Similar behavior has been previously observed by Cai et al. (2009) in *P. infestans* infected by PiRV-1. The latter authors also reported difficulties in curing PiRV-1 from host isolates, as well as the inability of transferring this virus *via* somatic fusion to a virus-free isolate.

All six viruses detected in *P. castaneae* are targeted by the *P. castaneae* RNAi machinery, and the most abundant size of sRNA reads in all four isolates was 21 nts, consistent with other *Phytophthora* species (Fahlgren et al., 2013; Jia et al., 2017; Botella and Jung, 2021) and the oomycete *Hyaloperonospora arabidopsidis* (Gäum.) Göker, Riethm., Voglmayr, Weiß & Oberw., a natural, obligate biotrophic downy mildew pathogen of *Arabidopsis thaliana* (L.) Heynh. (Dunker et al., 2020). In *P. condilina*, *P. infestans*, *P. ramorum*, *P. sojae* Kaufm. & Gerd., and *H. arabidopsidis*, besides the 21-nts sRNAs, the next most abundant sRNA population had size of 25-nts (Fahlgren et al., 2013; Dunker et al., 2020; Botella and Jung, 2021). In *P. parasitica*, two distinct types of sRNAs were 25–26 nts and 21 nts, but the 25–26 nts profiles were predominant (Jia et al., 2017). 21-nts sRNAs seem to derive primarily from inverted repeats, including a novel conserved microRNA family, several gene families, and Crinkler effectors (Fahlgren et al., 2013). Although it was demonstrated in this study that sRNA sequencing could be used to detect novel and relatively divergent viral species, difficulties in detecting the presence of PcaRV4 in *P. castaneae* isolates VN999, VN1008, and VN1004 by BlastX analyses suggest that the detection of novel virus species sharing a very low percentage of similarity with previously described viruses could be challenging. Therefore, it is necessary to have a more specific database. In cases where known viruses are expected, as shown, for example, in the wood rot fungus *Heterobasidion annosum sensu lato* (Vainio et al., 2015b), and in the pine pitch canker pathogen, *Fusarium circinatum* Nirenberg & O’Donnell infected by mitoviruses (Adalia et al., 2018), HTS of sRNAs can be used as a successful tool for virus diagnosis. If the genomes are not available and novel viruses show low levels of similarity to known viral species, such sequencing methods cannot be used alone for assembling long contigs or nearly complete genomes as shown in *P. condilina* (Botella and Jung, 2021) and the present study. Such findings indicate that sRNA seq is not the best technique for describing novel viral species divergent from previously described viruses due to short contig lengths. However, sRNA reads efficiently provided good coverage and high read numbers (Table 2) when mapped to the contigs obtained through the *de novo* assembly of total RNA reads, as demonstrated before (Botella and Jung, 2021).

The isolates used in this study originated from a *Chamaecyparis*-*Quercus* forest stand on Sau-Chua mountain in northern Vietnam, where *P. castaneae* was the only *Phytophthora* species recovered (Jung et al., 2020). Based on the sequences obtained through RT-PCR and Sanger sequencing of almost the entire length of the final viral contigs, the viruses PcaRV1-PcaRV4 and PcaNSRV1 show very low intraspecific variability in each of the four isolates. This might be explained by the homothallic mainly inbreeding sexual system of *P. castaneae* and its clonal mode of spread *via* asexual zoospores (Erwin and Ribeiro, 1996). Similarly, the intraspecific genetic variability of mitoviruses and partitiviruses infecting *Gremmeniella abietina* in one Spanish pine stand was very low (Botella et al., 2012, 2015). In *Heterobasidion parviporum,* a study showed that during persistent viral infections, single point mutations accumulate, resulting in virus diversification and occurrence of nearly identical viral sequence variants within single host clones (Vainio et al., 2015b).

In conclusion, this study represents the first evidence of multiple viral infections in the tree pathogen *P. castaneae* collected from its natural montane habitat in Vietnam. Sequencing of total stranded RNA was proven to be a powerful tool for detecting and *de novo* assembling of novel viral species and their genomes. The RNAi mechanism appears to actively target all six reported mycoviruses here, and their implications in the virulence of *P. castaneae* should be further investigated.

## Data Availability Statement

The RNA-Seq raw datasets presented in this study can be found in the online repository Sequence Read Archive (SRA), and they are available at this link https://www.ncbi.nlm.nih.gov/sra/PRJNA818851 (BioProject ID: PRJNA818851). The sequences of virus genomes are available in the NCBI GenBank database under the accession numbers listed in Table 1 of this manuscript.

## Author Contributions

MR, LB, and TJ: original idea and funding. AE, EH, EV, MR, LB, and SS: formal analysis and investigation. AE, EV, and MR: bioinformatics sRNA. MR and SS: bioinformatics of total RNA. TJ provided *P. castaneae* isolates. MR prepared the original draft and wrote the manuscript. LB contributed to the writing. All authors have read, critically reviewed, and edited the manuscript and agreed to the published version of the manuscript.

## Funding

This research was supported by the European Regional Development Fund, project “Phytophthora Research Centre,” Reg. No. CZ.02.1.01/0.0/0.0/15_003/0000453 and Specific University Research Fund of the Faculty of Forestry and Wood Technology, Mendel University in Brno LDF_VP_2019016.

## Conflict of Interest

The authors declare that the research was conducted in the absence of any commercial or financial relationships that could be construed as a potential conflict of interest.

## Publisher’s Note

All claims expressed in this article are solely those of the authors and do not necessarily represent those of their affiliated organizations, or those of the publisher, the editors and the reviewers. Any product that may be evaluated in this article, or claim that may be made by its manufacturer, is not guaranteed or endorsed by the publisher.

## Footnotes

^1^ http://hannonlab.cshl.edu/fastx_toolkit/

^2^ https://github.com/dzerbino/velvet/tree/master/contrib/AssemblyAssembler1.3

^3^ https://blast.ncbi.nlm.nih.gov

^4^ https://www.ncbi.nlm.nih.gov/orffinder/

^5^ https://www.ncbi.nlm.nih.gov/Structure/cdd/wrpsb.cgi

^6^ https://dotknot.csse.uwa.edu.au/

^7^ https://talk.ictvonline.org/
