# Supplementary material for: High Diversity of Novel Viruses in the Tree Pathogen Phytophthora castaneae Revealed by High-Throughput Sequencing of Total and Small RNA
Source: Front Microbiol. 2022 Jun 16;13:911474. doi: 10.3389/fmicb.2022.911474 (PMC9244493; doi:10.3389/fmicb.2022.911474)
Supplement: Supplementary file 1 [file Table_1.DOCX]

Supplementary Material

# Supplementary Tables

**Supplementary table 1.** Detailed information of virus specific primers used in detection and confirmation of viruses

| Acronym | Primer | Primer orientation | Primer Sequence 5’-3’ | Length | Position in the viral genome | Amplicon size (bp) | GC (%) | Tm (°C) |
| --- | --- | --- | --- | --- | --- | --- | --- | --- |
| PcaNSRV1 | Pc_bunya-like_1R | reverse | GCCTCCGCTATCATCACTGT | 20 | 4068-4087 | 552 | 50.0 | 59.6 |
| PcaNSRV1 | Pc_bunya-like_1F | forward | TCTGAGCCGACCAAAGTTGT | 20 | 4600-4619 | 552 | 50.0 | 59.5 |
| PcaRV1 | Megabirna_virus_RV | reverse | TGAACCTGGCTAAGCTGTGG | 20 | 2763-2782 | 568 | 55.0 | 60.0 |
| PcaRV1 | Megabirna_virus_FW | forward | GACCCATTCCCTCGAAAGCA | 20 | 3311-3330 | 568 | 55.0 | 60.0 |
| PcaRV2 | Pc_narna-like_1R | reverse | CATCGATCTGGTCTACGGGC | 20 | 1864-1883 | 619 | 60.0 | 60.0 |
| PcaRV2 | Pc_narna-like_1F | forward | CACCCTTCGGCTTACAGTGT | 20 | 1265 1284 | 619 | 55.0 | 60.0 |
| PcaRV3 | DN94529_i1_R | reverse | AGAGTGCCAGACCGAGATCT | 20 | 1821-1840 | 451 | 55.0 | 60.0 |
| PcaRV3 | DN94529_i1_F | forward | CTGGTCCTGTCGTCTTCACC | 20 | 1390-1409 | 451 | 60.0 | 60.0 |
| PcaRV4 | Pc_fusagra-like_3R | reverse | CATGATCACCAAGCCACCGA | 20 | 5363-5382 | 632 | 55.0 | 60.4 |
| PcaRV4 | Pc_fusagra-like_3F | forward | TAGCCGTTGTACACCACTGC | 20 | 4751-4770 | 632 | 55.0 | 60.3 |
| PcaRV5 | node139_VN8_endorna_1F | forward | TGTTCCAACTGACATTTCGGC | 21 | 124- 144 | 1048 | 47.6 | 59.4 |
| PcaRV5 | node295_VN8_endorna_1R | reverse | AGGTCTAGCGTACATTGGGT | 20 | 1,152-1,171 | 1048 | 50 | 58.1 |
| PcaRV5 | node_295-274_VN8_endornaF | forward | ACCCAATGTACGCTAGACCTTC | 22 | 1,152-1,173 | 853 | 50 | 59.8 |
| PcaRV5 | node_274-295_VN8_endornaR | reverse | TTATCATCTCCAAGCACGTATATG | 24 | 1,981- 2,004 | 853 | 37.5 | 56.5 |
| PcaRV5 | node274_VN8_endorna_2 F | forward | TCGGTGGAAGTGGAAAGGTC | 20 | 1,834- 1,853 | 345 | 55 | 59.6 |
| PcaRV5 | node351_VN8_endorna_2R | reverse | CGAAGACGCTTGAAATGTGGA | 21 | 2,158- 2,178 | 345 | 47.6 | 59.2 |
| PcaRV5 | node139_VN8_endorna_4F | forward | CGTGTGCTAAGTGTAGGCC | 19 | 5-23 | 243 | 57.9 | 58.2 |
| PcaRV5 | node_139-295_VN8_endornaR | reverse | CGCCTCGTTGTCACTTGAG | 19 | 229-247 | 243 | 57.9 | 58.9 |

**Supplementary Table 2** Detailed information of virus specific primers used in obtaining longer virus sequences across viral genomes

| Acronym | Primer name | Primer orientation | Primer Sequence 5’-3’ | Length | Position in the viral genome | Amplicon size (bp) | GC% | Tm (°C) |
| --- | --- | --- | --- | --- | --- | --- | --- | --- |
| PcaNSRV1 | Pc_bunya-like_1F | forward | TCTGAGCCGACCAAAGTTGT | 20 | 3,727 - 3,746 (4,619 - 4,600) | 552 | 50 | 59.5 |
| PcaNSRV1 | Pc_bunya-like_1R | reverse | GCCTCCGCTATCATCACTGT | 20 | 4,278 - 4,259 (4,068 - 4,087) | 552 | 55 | 59.6 |
| PcaNSRV1 | PcaNSRV1_1,222R | reverse | AGGCAGTCTCGTCTTGATTG | 20 | 1,222 - 1,203 (7,124 - 7,143) | 897 | 50 | 57.6 |
| PcaNSRV1 | PcaNSRV1_1,666F | forward | AGTGCCAAAAATAAAGAAATGCCA | 24 | 1,666 - 1,689 (6,680 - 6,657) | 839 | 33.3 | 58.4 |
| PcaNSRV1 | PcaNSRV1_1,730R | reverse | ACCTTCTCCACTTGCTCAGA | 20 | 1,730 - 1,711 (6,616 - 6,635) | 736 | 50 | 58.3 |
| PcaNSRV1 | PcaNSRV1_125F | reverse | TGTCATCCTCTAACAAGTCTATTGC | 25 | 8,221 - 8,197 (125 - 149) | 589 | 40 | 58.7 |
| PcaNSRV1 | PcaNSRV1_2,450F | forward | TTGCACCGCTGAAGAGAACT | 20 | 2,450 - 2,469 (5,896 - 5,877) | 741 | 50 | 59.9 |
| PcaNSRV1 | PcaNSRV1_2,504R | reverse | AGCCTTGAGACGAATTGCAG | 20 | 2,504 - 2,485 (5,842 - 5,861) | 839 | 50 | 58.6 |
| PcaNSRV1 | PcaNSRV1_3,118F | forward | ACAACTTCGACCTGTGTGCA | 20 | 3,116 - 3,135 (5,230 - 5,211) | 680 | 50 | 60.1 |
| PcaNSRV1 | PcaNSRV1_3,193R | reverse | CATTCACCCGGTCAAAAGGC | 20 | 3,190 - 3,171 (5,156 - 5,175) | 741 | 55 | 59.8 |
| PcaNSRV1 | PcaNSRV1_3,798R | reverse | TGGCGTAAAATGACACAGATATGAG | 25 | 3,795 - 3,771 (4,551 - 4,575) | 680 | 40 | 59.5 |
| PcaNSRV1 | PcaNSRV1_326F | forward | GCTTGGCTGACTTCACATTCA | 21 | 326 - 346 (8,020 - 8,000) | 897 | 47.6 | 59.1 |
| PcaNSRV1 | PcaNSRV1_4,129F | forward | GAATTCACAATGCCCTACAGCT | 22 | 4,126 - 4,147 (4,220 - 4,199) | 4809 | 45.5 | 59 |
| PcaNSRV1 | PcaNSRV1_4,839F | forward | TTACAAACATCACAAACTGACGG | 23 | 4,836 - 4,858 (3,510 - 3,488) | 924 | 39.1 | 57.8 |
| PcaNSRV1 | PcaNSRV1_4,937R | reverse | GCTTGACCCAGCGTAGTGTT | 20 | 4,934 - 4,915 (3,412 - 3,431) | 809 | 55 | 60.6 |
| PcaNSRV1 | PcaNSRV1_5,511F | forward | TGAGCGGTTTTGGACATACA | 20 | 5,505 - 5,524 (2,841 - 2,822) | 965 | 45 | 57.4 |
| PcaNSRV1 | PcaNSRV1_5,765R | reverse | ACTTCTTCCACCATGCCCAA | 20 | 5,759 - 5,740 (2,587 - 2,606) | 924 | 50 | 59.5 |
| PcaNSRV1 | PcaNSRV1_6,374F | forward | AGCTGGACCCAACACACAA | 19 | 6,368 - 6,386 (1,978 - 1,960) | 936 | 52.6 | 59.4 |
| PcaNSRV1 | PcaNSRV1_6,475R | reverse | CCATCACCAGTGCCCTAGTT | 20 | 6,469 - 6,450 (1,877 - 1,896) | 965 | 55 | 59.4 |
| PcaNSRV1 | PcaNSRV1_7,218F | forward | AAGGAAACAAGGGAGCGATT | 20 | 7,212 - 7,231 (1,134 - 1,115) | 749 | 45 | 57.4 |
| PcaNSRV1 | PcaNSRV1_7,309R | reverse | AGTCGTCACTGTCTTCATCTAGT | 23 | 7,303 - 7,281 (1,043 - 1,065) | 936 | 43.5 | 58.7 |
| PcaNSRV1 | PcaNSRV1_7,699F | reverse | GCATCGGGTGAAAAGGTATCATG | 23 | 7,960 - 7,940 (386 - 406) | 749 | 47.8 | 60 |
| PcaNSRV1 | PcaNSRV1_725R | forward | CGACGTTTTGAATCTCGCCC | 20 | 7,621 - 7,640 (725 - 706) | 589 | 55 | 59.9 |
| PcaNSRV1 | PcaNSRV1_995F | forward | TTAGGTCAAAGGGTGGGAAC | 20 | 995 - 1,014 (7,351 - 7,332) | 736 | 50 | 57 |
| PcaRV1 | PcaRV1_3660F | forward | GTGGCTTGCTTTCGAGGGAA | 20 | 3,652 - 3,671 | 758 | 55 | 60.9 |
| PcaRV1 | PcaRV1_5,601F | forward | CGCAGTACAGAAGTTTGAGGA | 21 | 5,585 - 5,605 | 723 | 47.6 | 58 |
| PcaRV1 | PcaRV1_6,334R | reverse | AGGAAGGGGGAGGACTGTAC | 20 | 6,317 - 6,298 | 723 | 55 | 59.4 |
| PcaRV1 | Megabirna_virus_ FW | reverse | GACCCATTCCCTCGAAAGCA | 20 | 3658 |  | 55 | 60 |
| PcaRV1 | PcaRV1_1,709F | forward | CTGCAAACCTGGCTTCTGTG | 20 | 1,709 - 1,728 | 865 | 55 | 59.7 |
| PcaRV1 | PcaRV1_1,803R | reverse | CTCTGTGGTGGTCAAGGAGC | 20 | 1,803 - 1,784 | 802 | 60 | 60.3 |
| PcaRV1 | PcaRV1_2,525F | forward | GCGAGCTGGATGAGACTCTC | 20 | 2,519 - 2,538 | 1159 | 60 | 60 |
| PcaRV1 | PcaRV1_2,573R | reverse | CTGACAGCACCCATGACGT | 19 | 2,573 - 2,555 | 865 | 57.9 | 60 |
| PcaRV1 | PcaRV1_998 | forward | CACTCCCGTCTACTTCAGGC | 20 | 998 - 1,017 | 802 | 60 | 59.8 |
| PcaRV1 | PcaRV1_1,117 | reverse | GTCCAGTCTGCCGCTCCT | 18 | 1,117 - 1,100 (1,115 -> 1,098) | 800 | 66.7 | 61.4 |
| PcaRV1 | PcaRV1_316F | forward | ACTGCGAGGGTCATAAAAGGT | 21 | 316 - 336 | 800 | 47.6 | 59.4 |
| PcaRV1 | PcaRV1_4,371F | forward | CTGGAAGGGGAAGGAGATGC | 20 | 4,361 - 4,380 | 685 | 60 | 59.8 |
| PcaRV1 | PcaRV1_4,419R | reverse | CCAGAGGGCTTTGGTAGGT | 19 | 4,409 - 4,391 | 758 | 57.9 | 58.6 |
| PcaRV1 | PcaRV1_4,973F | forward | CAAATGCTACGGTCTGGTGG | 20 | 4,973 - 4,992 | 797 | 55 | 58.9 |
| PcaRV1 | PcaRV1_5,057R | reverse | GTCCCCCAAATACCGCATCT | 20 | 5,057 - 5,038 | 685 | 55 | 59.8 |
| PcaRV1 | PcaRV1_5,601F | forward | CGCAGTACAGAAGTTTGAGGA | 21 | 5,601 - 5,621 | 733 | 47.6 | 58 |
| PcaRV1 | PcaRV1_5,773R | reverse | TGCCCTCATAGTACGCAACC | 20 | 5,773 - 5,754 | 797 | 55 | 59.8 |
| PcaRV1 | PcaRV1_6,334R | reverse | GGATCCCCAGGTCATGTTGT | 20 | 6,334 - 6,315 | 733 | 55 | 59.4 |
| PcaRV2 | Pc_narna-like_1R | reverse | CATCGATCTGGTCTACGGGC | 20 | 1,883 - 1,864 | 753 | 60 | 60 |
| PcaRV2 | PcaR2_238F | forward | CGCCGCTTTCGTATTTTCCTT | 21 | 200 - 220 | 935 | 47.6 | 59.9 |
| PcaRV2 | PcaRV2_1,169F | forward | GAATCCCTCTTCCTATACGCCA | 22 | 1,131 - 1,152 | 753 | 50 | 59.1 |
| PcaRV2 | PcaRV2_1,191R | reverse | CTGGCGTATAGGAAGAGGGATT | 22 | 1,153 - 1,132 | 935 | 50 | 59.1 |
| PcaRV2 | PcaRV2_1,756F | forward | GTCGGCCCTATCGGTTATCT | 20 | 1,718 - 1,737 | 671 | 55 | 58.7 |
| PcaRV2 | PcaRV2_2,290F | forward | ACTCTTACTTGGAACTATCTTCTCG | 25 | 2,251 - 2,275 | 443 | 40 | 57.9 |
| PcaRV2 | PcaRV2_2,427R | reverse | AACCAATCCCGCACACTG | 18 | 2,388 - 2,371 | 671 | 55.6 | 57.9 |
| PcaRV2 | PcaRV2_2,732R | reverse | GGTTAATGAGTAAAACTTGCTTTGC | 25 | 2,693 - 2,669 | 443 | 36 | 57.9 |
| PcaRV2 | PcaRV2_2,913R | reverse | GCGTCAATCCAAAGGTGCATT | 21 | 2,874 - 2,854 | 624 | 47.6 | 60.1 |
| PcaRV3 | DN94529_i1_R | reverse | AGAGTGCCAGACCGAGATCT | 20 | 1,840 - 1,821 | 947 | 55 | 60 |
| PcaRV3 | PcaRV3_2,241F | forward | GGCTCTCTCTCACACCTTGT | 20 | 2,219 - 2,238 | 851 | 55 | 59 |
| PcaRV3 | PcaRV3_2,448R | reverse | GCCTCCCGAAGAAAACACCA | 20 | 2,426 - 2,407 | 820 | 55 | 60.5 |
| PcaRV3 | PcaRV3_3,024F | forward | CCACACGGTAAGTCTGAAGC | 20 | 3,002 - 3,021 | 672 | 55 | 58.6 |
| PcaRV3 | PcaRV3_3,091R | reverse | TCACGCATAACACCAAACGC | 20 | 3,069 - 3,050 | 851 | 50 | 59.8 |
| PcaRV3 | PcaRV3_3,593F | forward | ACTGTGTGAGAGTTCGTGCG | 20 | 4,532 - 4,513 | 963 | 55 | 60.6 |
| PcaRV3 | PcaRV3_3,696R | reverse | TGCCGCCTCTCCTATCGAA | 19 | 3,673 - 3,655 | 672 | 57.9 | 60.5 |
| PcaRV3 | PcaRV3_4,504F | forward | CGGAGCTATCGTTCAGTCCT | 20 | 4,481 - 4,500 | 769 | 55 | 59 |
| PcaRV3 | PcaRV3_5,292R | reverse | AGCCAGTCCTCGCCAATAA | 19 | 5,269 - 5,251 | 769 | 52.6 | 58.7 |
| PcaRV3 | PcaRV3_916F | forward | CCGGAAATCACGTTGTCAGC | 20 | 894 - 913 | 947 | 55 | 59.8 |
| PcaRV3 | PcaRV3_1,629F | forward | CTTCTTGCACACGGAGGT | 18 | 1,607 - 1,624 | 820 | 55.6 | 57.2 |
| PcaRV4 | PcaRV4_1,010F | forward | TTCCCCACTTTTCCGTCGTT | 20 | 1,010 - 1,029 | 934 | 50 | 59.8 |
| PcaRV4 | PcaRV4_1,118R | reverse | ATGTCACCGCCCATCATCAT | 20 | 1,118 - 1,099 | 899 | 50 | 59.5 |
| PcaRV4 | PcaRV4_1,750F | forward | TTCAAGGTGTGGCTCGACTT | 20 | 1,750 - 1,769 | 428 | 50 | 59.5 |
| PcaRV4 | PcaRV4_1,923R | reverse | TAAAAGAACTCGTCACCCCGG | 21 | 1,943 - 1,923 | 934 | 52.4 | 60 |
| PcaRV4 | PcaRV4_2,135F | forward | TGCTCTCGTCGTACCACCT | 19 | 2,133 - 2,151 | 1095 | 57.9 | 60.3 |
| PcaRV4 | PcaRV4_2,179R | reverse | CTATCAGCAGTCTCGAAGCCA | 21 | 2,177 - 2,157 | 428 | 52.4 | 59.6 |
| PcaRV4 | PcaRV4_220F | forward | ATGTGTGTATGTGCCAGCGA | 20 | 220 - 239 | 899 | 50 | 60 |

**Supplementary Table 3.** The total number of contigs per isolate produced by *de novo* assembly of small RNA raw reads and information about minimum and a maximum length of contigs.

| Isolate name | Total number of contigs | Minimum length | Maximum length |
| --- | --- | --- | --- |
| VN999 | 747 | 68 | 513 |
| VN1004 | 891 | 52 | 782 |
| VN1008 | 455 | 57 | 1221 |
| VN1012 | 827 | 45 | 453 |

**Supplementary table 4** The total number of contigs per isolate produced by de novo assembly of small RNA raw reads and information about minimum and maximum length of contigs in isolate VN999.

| Contig number | Length | VN999 - BlastX first hit^2^ | QC(%)^3^ | E value | I (%)^4^ |
| --- | --- | --- | --- | --- | --- |
| Contig 14 | 144 | RdRp Halophytophthora RNA virus 6 (QLF99173.1) | 100% | 4e-12 | 56.25% |
| Contig 27 | 120 | polyprotein [Phytophthora cactorum bunyavirus 1] | 92% | 3e-14 | 89.19% |
| Contig 30 | 78 | polyprotein [Phytophthora cactorum bunyavirus 1] | 96% | 7e-06 | 68.00% |
| Contig 61 | 80 | RdRp Halophytophthora RNA virus 6 (QLF99173.1) | 97% | 2e-06 | 76.92% |
| Contig 62 | 71 | polyprotein [Phytophthora cactorum bunyavirus 1] | 97% | 2e-04 | 82.61% |
| Contig 84 | 371 | RdRp Rhizoctonia solani dsRNA virus 10 (QDW81304.1) | 57% | 7e-10 | 47.89% |
| Contig 106 | 83 | RdRp Halophytophthora RNA virus 6 (QLF99173.1) | 93% | 1e-08 | 73.08% |
| Contig 127 | 117 | RdRp Halophytophthora RNA virus 6 (QLF99173.1) | 97% | 1e-14 | 78.95% |
| Contig 140 | 75 | RdRp Pythium polare RNA virus 1 (YP_009552275.1) | 96% | 4e-07 | 91.67% |
| Contig 157 | 75 | putative RdRp [Totiviridae sp.] (UHS72561.1) | 84% | 6e-04 | 90.48% |
| Contig 169 | 74 | RdRp [Sanya narnavirus 11] (UHM27569.1) | 72% | 6e-05 | 88.89% |
| Contig 184 | 79 | RdRp [Phytophthora condilina negative stranded RNA virus 5] (QTT60997.1) | 94% | 1e-09 | 96.00% |
| Contig 186 | 108 | RdRp Rhizoctonia solani mycovirus 3 (ANR02704.1) | 100% | 1e-06 | 61.11% |
| Contig 188 | 74 | RdRp Halophytophthora RNA virus 6 (QLF99173.1) | 97% | 6e-09 | 91.67% |
| Contig 191 | 105 | RdRp Halophytophthora RNA virus 6 (QLF99173.1) | 97% | 2e-04 | 58.82% |
| Contig 210 | 111 | polyprotein [Phytophthora cactorum bunyavirus 1] (QUA12643.1) | 94% | 2e-05 | 57.14% |
| Contig 214 | 183 | RdRp Halophytophthora RNA virus 6 (QLF99173.1) | 85% | 2e-07 | 55.77% |
| Contig 218 | 102 | polyprotein [Phytophthora cactorum bunyavirus 1] | 97% | 4e-08 | 69.70% |
| Contig 235 | 120 | RdRp Halophytophthora RNA virus 6 (QLF99173.1) | 97% | 3e-13 | 76.92% |
| Contig 244 | 132 | polyprotein [Phytophthora cactorum bunyavirus 1] | 95% | 5e-10 | 57.14% |
| Contig 277 | 158 | RdRp Halophytophthora RNA virus 6 (QLF99173.1) | 93% | 7e-24 | 91.84% |
| Contig 370 | 139 | RdRp Halophytophthora RNA virus 6 (QLF99173.1) | 73% | 7e-11 | 85.29% |
| Contig 381 | 86 | putative RdRp [Totiviridae sp.] (UHS72506.1) | 97% | 0.005 | 57.14% |
| Contig 400 | 120 | polyprotein [Phytophthora cactorum bunyavirus 1] (QUA12643.1) | 92% | 2e-04 | 48.65% |
| Contig 423 | 110 | RdRp Halophytophthora RNA virus 6 (QLF99173.1) | 95% | 7e-06 | 65.71% |
| Contig 457 | 99 | putative RdRp [Totiviridae sp.] (UHS72506.1) | 90% | 0.031 | 63.33% |
| Contig 505 | 102 | RdRp Pythium polare RNA virus 1 (YP_009552275.1) | 76% | 5e-06 | 84.62% |
| Contig 546 | 68 | putative RdRp [Totiviridae sp.] (UHS72506.1) | 97% | 4e-04 | 81.82% |
| Contig 557 | 84 | RdRp Halophytophthora RNA virus 6 (QLF99173.1) | 96% | 2e-06 | 74.07% |
| Contig 678 | 88 | putative RdRp [Totiviridae sp.] (UHS72561.1) | 95% | 2e-04 | 71.43% |

The date of the last BlastX search 28.03.2022.

**Supplementary table 5.** The total number of contigs per isolate produced by de novo assembly of small RNA raw reads and information about minimum and maximum length of contigs in isolate VN1004.

| Contig number | Length | VN1004 BlastX first hit^2^ | QC [%]^3^ | E value | I [%]^4^ |
| --- | --- | --- | --- | --- | --- |
| Contig 17 | 129 | [polyprotein [Phytophthora cactorum bunyavirus 1] (QUA12643.1)](https://blast.ncbi.nlm.nih.gov/Blast.cgi#alnHdr_QUA12643) | 97 | 1.00E-06 | 54.76 |
| Contig 19 | 385 | [RdRp [Phytophthora cactorum RNA virus 1] (QUE45737.1)](https://blast.ncbi.nlm.nih.gov/Blast.cgi#alnHdr_QUE45737) | 95 | 2.00E-59 | 77.05 |
| Contig 28 | 383 | CP-RdRp fusion protein [Phytophthora cactorum RNA virus 1] (QJS39952.1) | 99 | 2.00E-66 | 81.89 |
| Contig 29 | 782 | [putative coat protein [Phytophthora cactorum RNA virus 1] (QJS39953.1)](https://blast.ncbi.nlm.nih.gov/Blast.cgi#alnHdr_QJS39953) | 48 | 6.00E-50 | 70.63 |
| Contig 30 | 375 | [RdRp [Halophytophthora RNA virus 6] (QLF99173.1)](https://blast.ncbi.nlm.nih.gov/Blast.cgi#alnHdr_QLF99173) | 72 | 7.00E-32 | 60.44 |
| Contig 31 | 226 | ORF2 [Bremia lactucae associated fusagravirus 1] (QIP68010.1) | 96 | 0.043 | 31.51 |
| Contig 32 | 91 | [RdRp [Phytophthora cactorum RNA virus 1] (QUE45736.1)](https://blast.ncbi.nlm.nih.gov/Blast.cgi#alnHdr_QUE45736) | 85 | 0.005 | 69.23 |
| Contig 44 | 134 | [RdRp [Phytophthora condilina RNA virus 1] (QTT60989.1)](https://blast.ncbi.nlm.nih.gov/Blast.cgi#alnHdr_QTT60989) | 98 | 3.00E-04 | 44.68 |
| Contig 62 | 214 | RdRp [Phytophthora cactorum RNA virus 1] (QUE45737.1) | 99 | 3.00E-30 | 78.87 |
| Contig 65 | 86 | RdRp [Phytophthora cactorum RNA virus 1] (QUE45741.1) | 97 | 6.00E-05 | 71.43 |
| Contig 67 | 767 | putative coat protein [Phytophthora cactorum RNA virus 1] (QJS39953.1) | 99 | 7E-136 | 76.08 |
| Contig 71 | 169 | polyprotein [Phytophthora cactorum bunyavirus 2] (QUA12644.1) | 95 | 8.00E-07 | 46.30 |
| Contig 73 | 269 | RdRp [Halophytophthora RNA virus 6] (QLF99173.1) | 99 | 3.00E-34 | 68.54 |
| Contig 78 | 190 | [CP-RdRp fusion protein [Phytophthora cactorum RNA virus 1] (QJS39952.1)](https://blast.ncbi.nlm.nih.gov/Blast.cgi#alnHdr_QJS39952) | 97 | 1.00E-19 | 72.58 |
| Contig 79 | 92 | [RdRp [Halophytophthora RNA virus 6] (QLF99173.1)](https://blast.ncbi.nlm.nih.gov/Blast.cgi#alnHdr_QLF99173) | 91 | 7.00E-04 | 71.43 |
| Contig 81 | 84 | [RdRp [Phytophthora condilina negative stranded RNA virus 5 (QTT60997.1)](https://blast.ncbi.nlm.nih.gov/Blast.cgi#alnHdr_QTT60997) | 100 | 1.00E-06 | 78.57 |
| Contig 86 | 129 | [RdRp [Pythium polare RNA virus 1] (YP_009552275.1)](https://blast.ncbi.nlm.nih.gov/Blast.cgi#alnHdr_YP_009552275) | 97 | 2.00E-10 | 64.29 |
| Contig 90 | 206 | [polyprotein [Phytophthora cactorum bunyavirus 1] (QUA12643.1)](https://blast.ncbi.nlm.nih.gov/Blast.cgi#alnHdr_QUA12643) | 99 | 8.00E-20 | 58.82 |
| Contig 99 | 175 | [putative coat protein [Pythium polare RNA virus 1] (YP_009552274.1)](https://blast.ncbi.nlm.nih.gov/Blast.cgi#alnHdr_YP_009552274) | 78 | 0.010 | 41.30 |
| Contig 128 | 114 | [RdRp [Plasmodium vivax Narna-Like virus 1] (QHQ74301.1)](https://blast.ncbi.nlm.nih.gov/Blast.cgi#alnHdr_QHQ74301) | 97 | 2.00E-05 | 54.05 |
| Contig 130 | 116 | [RdRp [Halophytophthora RNA virus 6] (QLF99173.1)](https://blast.ncbi.nlm.nih.gov/Blast.cgi#alnHdr_QLF99173) | 95 | 1.00E-09 | 75.68 |
| Contig 154 | 84 | [RdRp [Pythium polare RNA virus 1] (YP_009552275.1)](https://blast.ncbi.nlm.nih.gov/Blast.cgi#alnHdr_YP_009552275) | 96 | 0.005 | 62.96 |
| Contig 157 | 341 | [polyprotein [Phytophthora cactorum bunyavirus 1] (QUA12643.1)](https://blast.ncbi.nlm.nih.gov/Blast.cgi#alnHdr_QLF99172) | 99 | 1.00E-40 | 61.95 |
| Contig 167 | 392 | [polyprotein [Sherlock virus] (QED21500.1)](https://blast.ncbi.nlm.nih.gov/Blast.cgi#alnHdr_QED21500) | 95 | 5.00E-08 | 29.60 |
| Contig 168 | 144 | [RdRp [Halophytophthora RNA virus 6] (QLF99173.1)](https://blast.ncbi.nlm.nih.gov/Blast.cgi#alnHdr_QLF99173) | 100 | 4.00E-19 | 77.08 |
| Contig 170 | 167 | [RdRp [Pythium polare RNA virus 1] (YP_009552275.1)](https://blast.ncbi.nlm.nih.gov/Blast.cgi#alnHdr_YP_009552275) | 97 | 1.00E-10 | 55.56 |
| Contig 178 | 242 | [putative coat protein [Phytophthora cactorum RNA virus 1] (QUA12639.1)](https://blast.ncbi.nlm.nih.gov/Blast.cgi#alnHdr_QJS39953) | 92 | 6.00E-38 | 88.00 |
| Contig 181 | 286 | [putative coat protein [Phytophthora cactorum RNA virus 1] (QUA12639.1)](https://blast.ncbi.nlm.nih.gov/Blast.cgi#alnHdr_QJS39953) | 81 | 1.00E-37 | 84.62 |
| Contig 182 | 130 | [RdRp [Pythium polare RNA virus 1] (YP_009552275.1)](https://blast.ncbi.nlm.nih.gov/Blast.cgi#alnHdr_YP_009552275) | 96 | 0.001 | 48.84 |
| Contig 191 | 207 | [RdRp [Halophytophthora RNA virus 6] (QLF99158.1)](https://blast.ncbi.nlm.nih.gov/Blast.cgi#alnHdr_QLF99158) | 100 | 8.00E-13 | 45.71 |
| Contig 207 | 309 | [putative coat protein [Phytophthora cactorum RNA virus 1] (QJS39953.1)](https://blast.ncbi.nlm.nih.gov/Blast.cgi#alnHdr_QJS39953) | 99 | 4.00E-44 | 83.33 |
| Contig 215 | 150 | [RdRp [Pythium polare RNA virus 1] (YP_009552275.1)](https://blast.ncbi.nlm.nih.gov/Blast.cgi#alnHdr_YP_009552275) | 98 | 0.001 | 42.86 |
| Contig 219 | 229 | [RdRp [Halophytophthora RNA virus 6] (QLF99173.1)](https://blast.ncbi.nlm.nih.gov/Blast.cgi#alnHdr_QLF99173) | 98 | 8.00E-33 | 78.67 |
| Contig 228 | 236 | [RdRp [Pythium polare RNA virus 1] (YP_009552275.1)](https://blast.ncbi.nlm.nih.gov/Blast.cgi#alnHdr_YP_009552275) | 99 | 8.00E-23 | 58.97 |
| Contig 231 | 164 | [CP-RdRp fusion protein [Phytophthora cactorum RNA virus 1] (QUE45743.1)](https://blast.ncbi.nlm.nih.gov/Blast.cgi#alnHdr_QJS39952) | 98 | 3.00E-21 | 81.48 |
| Contig 242 | 147 | [RdRp [Pythium polare RNA virus 1] (YP_009552275.1)](https://blast.ncbi.nlm.nih.gov/Blast.cgi#alnHdr_YP_009552275) | 79 | 2.00E-13 | 74.36 |
| Contig 268 | 94 | [polyprotein [Phytophthora cactorum bunyavirus 1] (QUA12643.1)](https://blast.ncbi.nlm.nih.gov/Blast.cgi#alnHdr_QUA12643) | 98 | 1.00E-08 | 67.74 |
| Contig 271 | 82 | [RdRp [Halophytophthora RNA virus 6] (QLF99173.1)](https://blast.ncbi.nlm.nih.gov/Blast.cgi#alnHdr_QLF99173) | 95 | 4.00E-08 | 84.62 |
| Contig 268 | 207 | [putative coat protein [Pythium polare RNA virus 1] (YP_009552274.1)](https://blast.ncbi.nlm.nih.gov/Blast.cgi#alnHdr_YP_009552274) | 98 | 2.00E-11 | 48.53 |
| Contig 271 | 194 | [RdRp [Halophytophthora RNA virus 6] (QLF99173.1)](https://blast.ncbi.nlm.nih.gov/Blast.cgi#alnHdr_QLF99173) | 98 | 5.00E-25 | 75.00 |
| Contig 277 | 150 | [CP-RdRp fusion protein [Phytophthora cactorum RNA virus 1] (QUA12638.1)](https://blast.ncbi.nlm.nih.gov/Blast.cgi#alnHdr_QJS39952) | 98 | 1.00E-17 | 79.59 |
| Contig 294 | 98 | [polyprotein [Phytophthora cactorum bunyavirus 1] (QUA12643.1)](https://blast.ncbi.nlm.nih.gov/Blast.cgi#alnHdr_QUA12643) | 97 | 2.00E-04 | 65.62 |
| Contig 295 | 109 | [RdRp [Halophytophthora RNA virus 6] (QLF99173.1)](https://blast.ncbi.nlm.nih.gov/Blast.cgi#alnHdr_QLF99173) | 93 | 5.00E-13 | 85.29 |
| Contig 300 | 160 | [RdRp [Halophytophthora RNA virus 6] (QLF99173.1)](https://blast.ncbi.nlm.nih.gov/Blast.cgi#alnHdr_QLF99173) | 99 | 5.00E-24 | 88.68 |
| Contig 316 | 154 | [RdRp [Phytophthora condilina negative stranded RNA virus 5 (QTT60997.1)](https://blast.ncbi.nlm.nih.gov/Blast.cgi#alnHdr_QTT60997) | 99 | 7.00E-07 | 45.10 |
| Contig 327 | 152 | [ORF2 [Bremia lactucae associated fusagravirus 1] (QIP68010.1)](https://blast.ncbi.nlm.nih.gov/Blast.cgi#alnHdr_QIP68010) | 78 | 6.00E-05 | 45.00 |
| Contig 345 | 122 | [RdRp [uncultured virus] (AGW51771.1)](https://blast.ncbi.nlm.nih.gov/Blast.cgi#alnHdr_AGW51771) | 88 | 3.00E-04 | 48.65 |
| Contig 372 | 135 | [RdRp [Halophytophthora RNA virus 6] (QLF99173.1)](https://blast.ncbi.nlm.nih.gov/Blast.cgi#alnHdr_QLF99173) | 97 | 2.00E-22 | 93.18 |
| Contig 394 | 197 | [CP-RdRp fusion protein [Pythium splendens RNA virus 1] (BBJ21451.1)](https://blast.ncbi.nlm.nih.gov/Blast.cgi#alnHdr_BBJ21451) | 62 | 0.006 | 51.22 |
| Contig 401 | 149 | [RdRp [Pythium polare RNA virus 1] (YP_009552275.1)](https://blast.ncbi.nlm.nih.gov/Blast.cgi#alnHdr_YP_009552275) | 98 | 5.00E-06 | 55.10 |
| Contig 418 | 214 | [RdRp [Pythium polare RNA virus 1] (YP_009552275.1)](https://blast.ncbi.nlm.nih.gov/Blast.cgi#alnHdr_YP_009552275) | 99 | 4.00E-19 | 57.75 |
| Contig 432 | 148 | [RdRp [Halophytophthora RNA virus 6] (QLF99173.1)](https://blast.ncbi.nlm.nih.gov/Blast.cgi#alnHdr_QLF99173) | 99 | 3.00E-20 | 77.55 |
| Contig 445 | 107 | [RdRp [Pythium polare RNA virus 1] (YP_009552275.1)](https://blast.ncbi.nlm.nih.gov/Blast.cgi#alnHdr_YP_009552275) | 95 | 5.00E-09 | 70.59 |
| Contig 446 | 106 | [RdRp [Pythium polare RNA virus 1] (YP_009552275.1)](https://blast.ncbi.nlm.nih.gov/Blast.cgi#alnHdr_YP_009552275) | 96 | 3.00E-08 | 64.71 |
| Contig 469 | 103 | [CP-RdRp fusion protein [Phytophthora cactorum RNA virus 1] (QJS39952.1)](https://blast.ncbi.nlm.nih.gov/Blast.cgi#alnHdr_QJS39952) | 96 | 2.00E-07 | 66.67 |
| Contig 477 | 118 | [RdRp [Phytophthora condilina negative stranded RNA virus 5 (QTT60997.1)](https://blast.ncbi.nlm.nih.gov/Blast.cgi#alnHdr_QTT60997) | 99 | 8.00E-09 | 64.10 |
| Contig 497 | 128 | [polyprotein [Phytophthora cactorum bunyavirus 1] (QUA12643.1)](https://blast.ncbi.nlm.nih.gov/Blast.cgi#alnHdr_QUA12643) | 93 | 7.00E-16 | 80.00 |
| Contig 517 | 209 | [polyprotein [Phytophthora cactorum bunyavirus 1] (QUA12643.1)](https://blast.ncbi.nlm.nih.gov/Blast.cgi#alnHdr_QUA12643) | 97 | 5.00E-04 | 44.12 |
| Contig 529 | 104 | [RdRp [Phytophthora cactorum RNA virus 1] (QUE45744.1)](https://blast.ncbi.nlm.nih.gov/Blast.cgi#alnHdr_QUE45744) | 98 | 2.00E-08 | 70.59 |
| Contig 532 | 101 | [RdRp [Halophytophthora RNA virus 6] (QLF99173.1)](https://blast.ncbi.nlm.nih.gov/Blast.cgi#alnHdr_QLF99173) | 95 | 0.032 | 46.88 |
| Contig 563 | 117 | [CP-RdRp fusion protein [Phytophthora cactorum RNA virus 1] (QJS39952.1)](https://blast.ncbi.nlm.nih.gov/Blast.cgi#alnHdr_QJS39952) | 84 | 0.002 | 48.48 |
| Contig 781 | 353 | [RdRp [Pterostylis sanguinea virus A] (AOX47548.1)](https://blast.ncbi.nlm.nih.gov/Blast.cgi#alnHdr_AOX47548) | 69 | 5.00E-12 | 43.90 |
| Contig 782 | 79 | [RdRp [Bremia lactucae associated fusagravirus 1] (QIP68009.1)](https://blast.ncbi.nlm.nih.gov/Blast.cgi#alnHdr_QIP68009) | 91 | 0.004 | 62.50 |
| Contig 783 | 102 | [CP-RdRp fusion protein [Phytophthora cactorum RNA virus 1] (QUA12638.1)](https://blast.ncbi.nlm.nih.gov/Blast.cgi#alnHdr_QJS39952) | 97 | 1.00E-12 | 90.91 |
| Contig 784 | 98 | RdRp [Rhizoctonia solani mycovirus 3] (ANR02704.1) | 94 | 3.00E-04 | 64.52 |
| Contig 785 | 169 | RdRp [Pythium polare RNA virus 1] (YP_009552275.1) | 94 | 9.00E-14 | 62.26 |
| Contig 795 | 100 | CP-RdRp fusion protein [Phytophthora cactorum RNA virus 1] (QJS39952.1) | 72 | 2.00E-05 | 87.50 |
| Contig 797 | 171 | RdRp [Bremia lactucae associated fusagravirus 1] (QIP68009.1) | 89 | 0.001 | 43.14 |
| Contig 798 | 173 | putative coat protein [Pythium polare RNA virus 1] (YP_009552274.1) | 97 | 2.00E-13 | 57.14 |
| Contig 813 | 97 | RdRp [Sanya narnavirus 11] (UHM27569.1) | 83 | 8.00E-05 | 63.64 |
| Contig 825 | 194 | RdRp [Pythium polare RNA virus 1] (YP_009552275.1) | 37 | 0.001 | 70.83 |
| Contig 842 | 153 | RdRp [Pythium polare RNA virus 1] (YP_009552275.1) | 96 | 2.00E-12 | 63.27 |
| Contig 848 | 97 | RdRp [Pythium polare RNA virus 1] (YP_009552275.1) | 98 | 1.00E-05 | 72.73 |
| Contig 873 | 77 | polyprotein [Phytophthora cactorum bunyavirus 1] (QUA12643.1) | 97 | 1.00E-04 | 68.00 |

The date of the last BlastX search 14.02.2022.

**Supplementary table 6.** The total number of contigs per isolate produced by de novo assembly of small RNA raw reads and information about minimum and maximum length of contigs in isolate VN1008.

| Contig number | Length | VN1008 BlastX first hit^2^ | QC [%]^3^ | E value | I [%]^4^ |
| --- | --- | --- | --- | --- | --- |
| Contig 1 | 226 | RdRp [Phytophthora cactorum RNA virus 1] (QUE45737.1) | 95 | 5.00E-33 | 76.39 |
| Contig 9 | 128 | CP-RdRp fusion protein [Phytophthora cactorum RNA virus 1] (QJS39952.1) | 98 | 8.00E-07 | 61.90 |
| Contig 37 | 207 | CP-RdRp fusion protein [Phytophthora cactorum RNA virus 1] (QUA12638.1) | 98 | 2.00E-21 | 93.94 |
| Contig 39 | 168 | [RdRp [Sanya endornavirus 1] (UHM27586.1)](https://blast.ncbi.nlm.nih.gov/Blast.cgi#alnHdr_UHM27586) | 98 | 2.00E-06 | 41.82 |
| Contig 46 | 143 | Phytophthora condilina negative stranded RNA virus 5 (QTT60997.1) | 96 | 2.00E-15 | 71.74 |
| Contig 50 | 89 | [CP-RdRp fusion protein [Phytophthora cactorum RNA virus 1] (QJS39952.1)](https://blast.ncbi.nlm.nih.gov/Blast.cgi#alnHdr_QJS39952) | 97 | 3.00E-08 | 82.76 |
| Contig 52 | 115 | [CP-RdRp fusion protein [Phytophthora cactorum RNA virus 1] (QJS39952.1)](https://blast.ncbi.nlm.nih.gov/Blast.cgi#alnHdr_QJS39952) | 93 | 8.00E-16 | 86.11 |
| Contig 63 | 82 | [polyprotein [Phytophthora cactorum bunyavirus 1] (QUA12643.1)](https://blast.ncbi.nlm.nih.gov/Blast.cgi#alnHdr_QUA12643) | 98 | 3.00E-05 | 74.07 |
| Contig 66 | 76 | [polyprotein [Phytophthora cactorum bunyavirus 1] (QUA12643.1)](https://blast.ncbi.nlm.nih.gov/Blast.cgi#alnHdr_QUA12643) | 98 | 0.005 | 68.00 |
| Contig 98 | 88 | RdRp [Halophytophthora RNA virus 6] (QLF99173.1) | 98 | 2.00E-11 | 89.66 |
| Contig 112 | 81 | [RdRp [Rhizoctonia solani dsRNA virus 18] (QXI69650.1)](https://blast.ncbi.nlm.nih.gov/Blast.cgi#alnHdr_QXI69650) | 92 | 3.00E-05 | 64.00 |
| Contig 139 | 147 | [polyprotein [Erysiphe cichoracearum alphaendornavirus] (YP_009225663.1)](https://blast.ncbi.nlm.nih.gov/Blast.cgi#alnHdr_YP_009225663) | 97 | 2.00E-07 | 50.00 |
| Contig 146 | 106 | [RdRp [Pythium polare RNA virus 1] (YP_009552275.1)](https://blast.ncbi.nlm.nih.gov/Blast.cgi#alnHdr_YP_009552275) | 93 | 6.00E-11 | 78.79 |
| Contig 148 | 74 | [RdRp [Pythium polare RNA virus 1] (YP_009552275.1)](https://blast.ncbi.nlm.nih.gov/Blast.cgi#alnHdr_YP_009552275) | 89 | 0.001 | 81.82 |
| Contig 162 | 107 | [RdRp [Halophytophthora RNA virus 6] (QLF99173.1)](https://blast.ncbi.nlm.nih.gov/Blast.cgi#alnHdr_QLF99173) | 78 | 2.00E-04 | 71.43 |
| Contig 183 | 85 | [CP-RdRp fusion protein [Phytophthora cactorum RNA virus 1] (QUA12638.1)](https://blast.ncbi.nlm.nih.gov/Blast.cgi#alnHdr_QJS39952) | 95 | 1.00E-05 | 77.78 |
| Contig 188 | 113 | [polyprotein [Phytophthora cactorum bunyavirus 2] (QUA12644.1)](https://blast.ncbi.nlm.nih.gov/Blast.cgi#alnHdr_QUA12644) | 98 | 0.050 | 48.65 |
| Contig 191 | 97 | [CP-RdRp fusion protein [Phytophthora cactorum RNA virus 1] (QJS39952.1)](https://blast.ncbi.nlm.nih.gov/Blast.cgi#alnHdr_QJS39952) | 77 | 3.00E-08 | 96.00 |
| Contig 209 | 119 | [RdRp [Halophytophthora RNA virus 6] (QLF99173.1)](https://blast.ncbi.nlm.nih.gov/Blast.cgi#alnHdr_QLF99173) | 98 | 2.00E-12 | 71.79 |
| Contig 215 | 105 | [polyprotein [Phytophthora cactorum bunyavirus 1] (QUA12643.1)](https://blast.ncbi.nlm.nih.gov/Blast.cgi#alnHdr_QUA12643) | 100 | 3.00E-12 | 88.57 |
| Contig 229 | 73 | [polyprotein [Phytophthora cactorum bunyavirus 1] (QUA12643.1)](https://blast.ncbi.nlm.nih.gov/Blast.cgi#alnHdr_QUA12643) | 98 | 0.026 | 62.50 |
| Contig 235 | 91 | [CP-RdRp fusion protein [Phytophthora cactorum RNA virus 1] (QUA12638.1)](https://blast.ncbi.nlm.nih.gov/Blast.cgi#alnHdr_QJS39952) | 98 | 8.00E-06 | 70.00 |
| Contig 240 | 79 | [RdRp [Halophytophthora RNA virus 6] ( QLF99173.1)](https://blast.ncbi.nlm.nih.gov/Blast.cgi#alnHdr_QLF99173) | 98 | 4.00E-07 | 88.46 |
| Contig 265 | 131 | [putative coat protein [Phytophthora cactorum RNA virus 1] (QUA12639.1)](https://blast.ncbi.nlm.nih.gov/Blast.cgi#alnHdr_QJS39953) | 89 | 5.00E-14 | 84.62 |
| Contig 274 | 69 | [polyprotein [Chalara endornavirus CeEV1] (ADN43901.1)](https://blast.ncbi.nlm.nih.gov/Blast.cgi#alnHdr_ADN43901) | 91 | 3.00E-05 | 76.19 |
| Contig 293 | 135 | [RdRp [Halophytophthora RNA virus 6] ( QLF99173.1)](https://blast.ncbi.nlm.nih.gov/Blast.cgi#alnHdr_QLF99173) | 97 | 3.00E-19 | 81.82 |
| Contig 295 | 173 | [RdRp [Sanya endornavirus 1] (UHM27586.1)](https://blast.ncbi.nlm.nih.gov/Blast.cgi#alnHdr_UHM27586) | 98 | 7.00E-09 | 43.86 |
| Contig 297 | 90 | [RdRp [Halophytophthora RNA virus 6] (QLF99173.1)](https://blast.ncbi.nlm.nih.gov/Blast.cgi#alnHdr_QLF99173) | 90 | 2.00E-04 | 62.96 |
| Contig 335 | 115 | [RdRp [Halophytophthora RNA virus 6] (QLF99173.1)](https://blast.ncbi.nlm.nih.gov/Blast.cgi#alnHdr_QLF99173) | 99 | 3.00E-12 | 71.05 |
| Contig 351 | 69 | [polyprotein [Chalara endornavirus CeEV1] (ADN43901.1)](https://blast.ncbi.nlm.nih.gov/Blast.cgi#alnHdr_ADN43901) | 100 | 4.00E-04 | 65.22 |
| Contig 430 | 76 | [RdRp [Phytophthora cactorum RNA virus 1] (QUE45732.1)](https://blast.ncbi.nlm.nih.gov/Blast.cgi#alnHdr_QUE45732) | 98 | 0.009 | 72.00 |

The date of the last BlastX search 15.02.2022.

**Supplementary table 7.** The total number of contigs per isolate produced by de novo assembly of small RNA raw reads and information about minimum and maximum length of contigs in isolate VN1012.

| Contig number | Length | VN1012 BlastX first hit^2^ | QC [%]^3^ | E value | I [%]^4^ |
| --- | --- | --- | --- | --- | --- |
| Contig 8 | 85 | RdRp [Halophytophthora RNA virus 6] (QLF99173.1) | 95 | 0.002 | 70.37 |
| Contig 12 | 230 | [RdRp [Halophytophthora RNA virus 6] (QLF99173.1)](https://blast.ncbi.nlm.nih.gov/Blast.cgi#alnHdr_QLF99173) | 99 | 7.00E-33 | 77.63 |
| Contig 15 | 74 | [CP-RdRp fusion protein [Phytophthora cactorum RNA virus 1] (QUA12638.1)](https://blast.ncbi.nlm.nih.gov/Blast.cgi#alnHdr_QJS39952) | 97 | 7.00E-07 | 91.67 |
| Contig 22 | 237 | [putative coat protein [Phytophthora cactorum RNA virus 1] (QUE45748.1)](https://blast.ncbi.nlm.nih.gov/Blast.cgi#alnHdr_QJS39953) | 93 | 2.00E-23 | 79.73 |
| Contig 24 | 228 | [RdRp [Halophytophthora RNA virus 6] (QLF99173.1)](https://blast.ncbi.nlm.nih.gov/Blast.cgi#alnHdr_QLF99173) | 98 | 2.00E-20 | 54.67 |
| Contig 25 | 268 | [polyprotein [Phytophthora cactorum bunyavirus 1] (QUA12643.1)](https://blast.ncbi.nlm.nih.gov/Blast.cgi#alnHdr_QUA12643) | 97 | 4.00E-32 | 58.62 |
| Contig 26 | 133 | [RdRp [Phytophthora condilina negative stranded RNA virus 5] (QTT60997.1)](https://blast.ncbi.nlm.nih.gov/Blast.cgi#alnHdr_QTT60997) | 99 | 4.00E-21 | 93.18 |
| Contig 27 | 102 | [putative coat protein [Phytophthora cactorum RNA virus 1] (QJS39953.1)](https://blast.ncbi.nlm.nih.gov/Blast.cgi#alnHdr_QJS39953) | 82 | 3.00E-06 | 82.14 |
| Contig 35 | 79 | [RdRp [Halophytophthora RNA virus 6] (QLF99173.1)](https://blast.ncbi.nlm.nih.gov/Blast.cgi#alnHdr_QLF99173) | 72 | 1.00E-05 | 89.47 |
| Contig 40 | 75 | [RdRp [Pythium polare RNA virus 1] (YP_009552275.1)](https://blast.ncbi.nlm.nih.gov/Blast.cgi#alnHdr_YP_009552275) | 80 | 0.007 | 90.00 |
| Contig 48 | 280 | [RdRp [Halophytophthora RNA virus 6] (QLF99173.1)](https://blast.ncbi.nlm.nih.gov/Blast.cgi#alnHdr_QLF99173) | 94 | 4.00E-14 | 76.74 |
| Contig 54 | 171 | [RdRp [Cotesia vestalis bracovirus] (AEE09607.1)](https://blast.ncbi.nlm.nih.gov/Blast.cgi#alnHdr_AEE09607) | 87 | 2.00E-07 | 48.00 |
| Contig 55 | 278 | [polyprotein [Phytophthora cactorum bunyavirus 1] (QUA12643.1)](https://blast.ncbi.nlm.nih.gov/Blast.cgi#alnHdr_QUA12643) | 99 | 1.00E-30 | 68.48 |
| Contig 66 | 412 | [CP-RdRp fusion protein [Phytophthora cactorum RNA virus 1] (QJS39952.1)](https://blast.ncbi.nlm.nih.gov/Blast.cgi#alnHdr_QJS39952) | 99 | 7.00E-54 | 68.38 |
| Contig 77 | 247 | [RdRp [Halophytophthora RNA virus 6] (QLF99173.1)](https://blast.ncbi.nlm.nih.gov/Blast.cgi#alnHdr_QLF99173) | 97 | 1.00E-23 | 52.50 |
| Contig 81 | 163 | [polyprotein [Phytophthora cactorum bunyavirus 1] (QUA12643.1)](https://blast.ncbi.nlm.nih.gov/Blast.cgi#alnHdr_QUA12643) | 95 | 3.00E-15 | 59.62 |
| Contig 90 | 93 | [polyprotein [Phytophthora cactorum bunyavirus 1] (QUA12643.1)](https://blast.ncbi.nlm.nih.gov/Blast.cgi#alnHdr_QUA12643) | 87 | 0.021 | 55.56 |
| Contig 95 | 453 | [CP-RdRp fusion protein [Phytophthora cactorum RNA virus 1] (QJS39952.1)](https://blast.ncbi.nlm.nih.gov/Blast.cgi#alnHdr_QJS39952) | 99 | 1.00E-80 | 82.67 |
| Contig 96 | 77 | [RdRp [Phytophthora cactorum bunyavirus 1] (QUE45703.1)](https://blast.ncbi.nlm.nih.gov/Blast.cgi#alnHdr_QUE45703) | 77 | 7.00E-04 | 90.00 |
| Contig 100 | 261 | [RdRp [Phytophthora cactorum RNA virus 1] (QUE45737.1)](https://blast.ncbi.nlm.nih.gov/Blast.cgi#alnHdr_QUE45737) | 98 | 6.00E-39 | 77.91 |
| Contig 112 | 72 | [RdRp [Plasmodium vivax Narna-Like virus 1] (QHQ74301.1)](https://blast.ncbi.nlm.nih.gov/Blast.cgi#alnHdr_QHQ74301) | 91 | 0.003 | 72.73 |
| Contig 114 | 190 | [putative DNA helicase [Cotesia vestalis bracovirus] (AEE09607.1)](https://blast.ncbi.nlm.nih.gov/Blast.cgi#alnHdr_AEE09607) | 99 | 7.00E-14 | 50.79 |
| Contig 125 | 89 | [polyprotein [Phytophthora cactorum bunyavirus 1] (QUA12643.1)](https://blast.ncbi.nlm.nih.gov/Blast.cgi#alnHdr_QUA12643) | 94 | 0.007 | 64.29 |
| Contig 129 | 74 | [RdRp [Halophytophthora RNA virus 6] (QLF99173.1)](https://blast.ncbi.nlm.nih.gov/Blast.cgi#alnHdr_QLF99173) | 97 | 7.00E-07 | 87.50 |
| Contig 131 | 129 | [RdRp [Halophytophthora RNA virus 6] (QLF99173.1)](https://blast.ncbi.nlm.nih.gov/Blast.cgi#alnHdr_QLF99173) | 97 | 3.00E-15 | 80.95 |
| Contig133 | 78 | [polyprotein [Phytophthora cactorum bunyavirus 1] (QUA12643.1)](https://blast.ncbi.nlm.nih.gov/Blast.cgi#alnHdr_QUA12643) | 96 | 3.00E-05 | 80.00 |
| Contig 137 | 321 | [RdRp [Phytophthora cactorum RNA virus 1] (QUE45743.1)](https://blast.ncbi.nlm.nih.gov/Blast.cgi#alnHdr_QUE45743) | 98 | 9.00E-47 | 77.14 |
| Contig 147 | 210 | [RdRp [Pythium polare RNA virus 1] (YP_009552275.1)](https://blast.ncbi.nlm.nih.gov/Blast.cgi#alnHdr_YP_009552275) | 88 | 3.00E-20 | 62.90 |
| Contig 149 | 93 | [RdRp [Halophytophthora RNA virus 6] (QLF99173.1)](https://blast.ncbi.nlm.nih.gov/Blast.cgi#alnHdr_QLF99173) | 100 | 2.00E-05 | 61.29 |
| Contig 156 | 81 | [RdRp [Phytophthora cactorum RNA virus 1] ( QUE45743.1)](https://blast.ncbi.nlm.nih.gov/Blast.cgi#alnHdr_QUE45743) | 96 | 7.00E-06 | 76.92 |
| Contig 159 | 108 | [RdRp [Halophytophthora RNA virus 6] (QLF99173.1)](https://blast.ncbi.nlm.nih.gov/Blast.cgi#alnHdr_QLF99173) | 100 | 1.00E-14 | 91.67 |
| Contig 174 | 150 | [RdRp [Hangzhou totivirus 6] (UHK03176.1)](https://blast.ncbi.nlm.nih.gov/Blast.cgi#alnHdr_UHK03176) | 90 | 0.048 | 40.00 |
| Contig 185 | 197 | [RdRp [Phytophthora cactorum RNA virus 1] (QUE45737.1)](https://blast.ncbi.nlm.nih.gov/Blast.cgi#alnHdr_QUE45737) | 98 | 4.00E-26 | 75.38 |
| Contig 195 | 133 | [CP-RdRp fusion protein [Phytophthora cactorum RNA virus 1] (QUA12638.1)](https://blast.ncbi.nlm.nih.gov/Blast.cgi#alnHdr_QJS39952) | 99 | 2.00E-16 | 79.55 |
| Contig 210 | 152 | [polyprotein [Phytophthora cactorum bunyavirus 1] (QUA12643.1)](https://blast.ncbi.nlm.nih.gov/Blast.cgi#alnHdr_QUA12643) | 96 | 7.00E-14 | 63.27 |
| Contig 215 | 137 | [RdRp [Pythium polare RNA virus 1] (YP_009552275.1)](https://blast.ncbi.nlm.nih.gov/Blast.cgi#alnHdr_YP_009552275) | 91 | 0.006 | 38.10 |
| Contig 220 | 103 | [CP-RdRp fusion protein [Phytophthora cactorum RNA virus 1] (QUA12638.1)](https://blast.ncbi.nlm.nih.gov/Blast.cgi#alnHdr_QJS39952) | 99 | 6.00E-12 | 85.29 |
| Contig 227 | 209 | [RdRp [Pythium polare RNA virus 1] (YP_009552275.1)](https://blast.ncbi.nlm.nih.gov/Blast.cgi#alnHdr_YP_009552275) | 55 | 5.00E-10 | 71.79 |
| Contig 233 | 170 | [RdRp [Phytophthora condilina negative stranded RNA virus 5] (QTT60997.1)](https://blast.ncbi.nlm.nih.gov/Blast.cgi#alnHdr_QTT60997) | 54 | 0.011 | 58.06 |
| Contig 243 | 134 | [CP-RdRp fusion protein [Phytophthora cactorum RNA virus 1] (QJS39952.1)](https://blast.ncbi.nlm.nih.gov/Blast.cgi#alnHdr_QJS39952) | 58 | 7.00E-05 | 73.08 |
| Contig 251 | 84 | [polyprotein [Phytophthora cactorum bunyavirus 1] (QUA12643.1)](https://blast.ncbi.nlm.nih.gov/Blast.cgi#alnHdr_QUA12643) | 92 | 6.00E-04 | 69.23 |
| Contig 294 | 163 | [RdRp [Pythium polare RNA virus 1] (YP_009552275.1)](https://blast.ncbi.nlm.nih.gov/Blast.cgi#alnHdr_YP_009552275) | 97 | 1.00E-13 | 60.38 |
| Contig 295 | 280 | [putative coat protein [Phytophthora cactorum RNA virus 1] (QUA12639.1)](https://blast.ncbi.nlm.nih.gov/Blast.cgi#alnHdr_QJS39953) | 67 | 2.00E-17 | 65.08 |
| Contig 345 | 104 | [RdRp [Pythium polare RNA virus 1] (YP_009552275.1)](https://blast.ncbi.nlm.nih.gov/Blast.cgi#alnHdr_YP_009552275) | 89 | 1.00E-09 | 77.42 |
| Contig 355 | 156 | [CP-RdRp fusion protein [Phytophthora cactorum RNA virus 1] (QJS39952.1)](https://blast.ncbi.nlm.nih.gov/Blast.cgi#alnHdr_QJS39952) | 98 | 3.00E-18 | 70.59 |
| Contig 372 | 137 | [RdRp [Pythium polare RNA virus 1] (YP_009552275.1)](https://blast.ncbi.nlm.nih.gov/Blast.cgi#alnHdr_YP_009552275) | 98 | 3.00E-12 | 64.44 |
| Contig 437 | 100 | [CP-RdRp fusion protein [Phytophthora cactorum RNA virus 1] (QJS39952.1)](https://blast.ncbi.nlm.nih.gov/Blast.cgi#alnHdr_QJS39952) | 75 | 0.002 | 72.00 |
| Contig 455 | 97 | [RdRp [Pythium polare RNA virus 1] (YP_009552275.1)](https://blast.ncbi.nlm.nih.gov/Blast.cgi#alnHdr_YP_009552275) | 95 | 6.00E-04 | 58.06 |
| Contig 469 | 85 | [RdRp [Pythium polare RNA virus 1] (YP_009552275.1)](https://blast.ncbi.nlm.nih.gov/Blast.cgi#alnHdr_YP_009552275) | 98 | 0.015 | 64.29 |
| Contig 487 | 76 | [RdRp [Pythium polare RNA virus 1] (YP_009552275.1)](https://blast.ncbi.nlm.nih.gov/Blast.cgi#alnHdr_YP_009552275) | 98 | 1.00E-05 | 72.00 |
| Contig 656 | 87 | [RdRp [Halophytophthora RNA virus 6] (QLF99173.1)](https://blast.ncbi.nlm.nih.gov/Blast.cgi#alnHdr_QLF99173) | 96 | 2.00E-09 | 85.71 |
| Contig 729 | 102 | RdRp [Rhizoctonia solani megabirnavirus 2] (QTF98696.1) | 97 | 5.00E-06 | 57.58 |
| Contig 734 | 159 | CP-RdRp fusion protein [Phytophthora cactorum RNA virus 1] (QJS39952.1) | 77 | 8.00E-17 | 80.49 |
| Contig 736 | 154 | RdRp [Pythium polare RNA virus 1] (YP_009552275.1) | 97 | 5.00E-13 | 64.71 |
| Contig 737 | 120 | [putative coat protein [Phytophthora cactorum RNA virus 1] (QUE45748.1)](https://blast.ncbi.nlm.nih.gov/Blast.cgi#alnHdr_QUE45748) | 97 | 2.00E-15 | 92.31 |
| Contig 741 | 166 | [putative RdRp [uncultured virus] (AGW51771.1)](https://blast.ncbi.nlm.nih.gov/Blast.cgi#alnHdr_AGW51771) | 86 | 5.00E-05 | 43.40 |
| Contig 767 | 92 | RdRp [Rhizoctonia solani mycovirus 3] (ANR02704.1) | 81 | 0.025 | 68.00 |
| Contig 773 | 86 | RdRp [Pythium polare RNA virus 1] (YP_009552275.1) | 83 | 3.00E-05 | 79.17 |
| Contig 774 | 143 | RdRp [Pythium polare RNA virus 1] (YP_009552275.1) | 90 | 3.00E-10 | 65.12 |
| Contig 787 | 77 | CP-RdRp fusion protein [Phytophthora cactorum RNA virus 1] (QUA12638.1) | 97 | 2.00E-07 | 96.00 |
| Contig 804 | 79 | RdRp [Halophytophthora RNA virus 6] (QLF99173.1) | 94 | 0.034 | 68.00 |

The date of the last BlastX search 15.02.2022.

**Supplementary table 8.** Statistics of Stranded RNA seq data of RNA pool containing RNA of isolate VN999

| Sample ID^1^ | Total read bases (bp)^2^ | Total Reads^3^ | GC (%)^4^ | AT(%)^5^ | Q20 (%)^6^ | Q30(%)^7^ |
| --- | --- | --- | --- | --- | --- | --- |
| VN999 | 12,860,573,006 | 127,332,406 | 53.071 | 46.93 | 98.015 | 94.273 |

^1^Sample name; ^2^Total number of bases sequenced; ^3^Total number of reads; ^4^The sum of read 1 and read 2; ^4^GC content; ^5^AT content; ^6^Ratio of bases that have phred quality score of over 20; ^7^Ratio of bases that have phred quality score of over 30.

**Supplementary table 9**. Validation of Trinity contigs by Sanger sequencing and detection of polymorphic sites/double peaks

| Virus acronym | Trinity contig length | Sanger seq length | Identity % | Polymorphic sites | Number of double peaks | Polymorphic sites (X) and occurrence of double peaks (chosen/alternative base) |
| --- | --- | --- | --- | --- | --- | --- |
| PcaNSRV1 | 8,345 | 7,861 | 99.949% | 4 | 4 | 466 (T/C), 1626 (T/C), 4341 (G/A), 7248 (A/G) |
| PcaRV1 | 6,337 | 5,985 | 100% | 0 | 0 | 0 |
| PcaRV2 | 2,891 | 2,644 | 98.828 % | 31 | 20 | 539 (T/C), 664 (C/T), 837 (X), 862 (T/C), 898 (G/A), 1012 (T/C), 1018 (G/A), 1039 (T/C), 1045 (A/G), 1174 (T/C), 1192 (A/T), 1234 (G/A), 1256 (C/T), 1289 (C/T), 1351 (G/A), 1393 (G/A), 1438 (X), 1472-1474 (X), 1492 (X), 1513 (X), 1591 (X), 1606 (G/A), 1660 (X), 1663 (X), 1684 (X), 1831 (A/G), 1918 (G/C), 2404 (T/G), 2591 (T/C). |
| PcaRV3 | 5,470 | 4,306 | 100% | 0 | 0 | 0 |
| PcaRV4 | 6,884 | 2,979 | 99.966 % | 1 | 1 | 721 (T/A) |

Sequences obtained by Sanger were mapped to the final Trinity contigs with Geneious mapper and Medium/Low sensitivity. Pairwise alignments of Trinity contigs and final Sanger sequences was done by MAFFT v7.450 in Geneious Prime® 2020.2.3

**Supplementary table 10**. The Trinity contigs originating from “cleaned" total RNA-seq reads of VN999 encoding for putative proteins showing low similarity in nr database of NCBI.

| Trinity Contig ID | Length (nt) | Reads^1^ | Coverage %^2^ | Protein length (aa)^3^ | M^r^ of the protein^4^ | Sequence of the predicted protein |
| --- | --- | --- | --- | --- | --- | --- |
| DN4299_c4_g1_i7 | 2,584 | 21,391 | 846.3 | 801 | 93.614 kDa | MKIFVTSKVSNTQDILYWTQRRMETEKWDIDKNILLNVSLMLSIFALGLMFAVTNYIFIATGLIGKVWNLRQESKNVDLISFYACMVGTLSLSQHSLTMAPLLLLPLGIYSYRATLVPLVELVKVSSATSVLIMMVSTVVSHSFGNFRALQVSLLIYNISISDKCTTKDARYLLLYKMLLNMGATSPCLQKYLKSPFSYFKTTENSKCYHVEPCLGLRAKTRLPKKILTVVLLSKWMDSQVNKLAGLDPVMTLLFGWLHTLGFYPPFIKPIAGVVTAARGKRRANMDVEKLAEWAVNKQNYVKTHDGVIRTLFLLYRQRFDKMSKKERKLAYQFSNIPNFRLTKAKVNALKMKILKTSSYLPAVFDINYKPYTSMVKVRIEKEYIKTPLTKEAERAWSSFRGCYTDDTKRVLIKETVLTQSGSCFFKMKSDITKKENFKEMRKKARSLTFLHSTKTMMTRNTNCVYRRKFKMQKKTVQDADLKSTFNKKIDPSKLRHFTKPLMMTQKVTCENIDTMRKVDYFHASLNYKLQEQAYYCNSSPNMVCDALKFVEGIRNLQKQNIETPPIQDHHYSGYIAEITAAAEKKKKLIEDARAELVGPVTSKRAPKREKKEMRRSLRKERKKKYKEDQKRKKEEMDEYGYYSMDEEERYGMADEDDGEDKLTFEELDEMYREPELDETARFKPGEWQPPKHVPLPKPPKVTVEEYQFPARYKKIIRFKAHEAQWLVRRKNRFLEKLEDTELKKFAPQITNWVVNATRTRMMSLWGMTTFAKPRNTFQTDLKEMKARLRMIEVCRLGSKK |
| DN86043_c0_g1_i1 | 1,161 | 162 | 14.3 | 105 | 11.174 kDa | MSRTSNQHAQHEARRAKPPAQESQQAKPTPSPASEAARRNVLTLTLLVALGRMPLGARSRSPSQPENCPGDVARPAAGPTISHPRLVSAFPPQLRSQPASPLPPH |
| DN87966_c0_g1_i2 | 814 | 153 | 18.9 | 201 | 22.332 kDa | MKQILIARISGKRQNFIPKFRCKCGLLLCCCCDREYRRRRTQSTALVQPLPLCHCPTPAGPDQAQNTEKSAKSKPAFHDAACGRATRDVTCNFRNCVVTRACHRMPKATRPQQRSYTQSTREPSSVRLLAVASSCGGYPSIDPLVSAMRVSDDPTQPWSATALSGRSRCYPSPDLQHGVGLLYALHFQQQQHKPTTGSRRA |
| DN90996_c0_g1_i1 | 1,448 | 103,275 | 7618.7 | 229 | 26.613 kDa | MKRSVTFFVPSTKVRTKPLNEWVLKVPTPKEKEPLGSVIDTAFCSFNKRAPHVYTVDVTKLSNEVRKIQPWDYLKVAGDCNASAETQAVLQSVSGTVRTLREQRIHATKSMAQGRVVWRKILSQMTNSLNKIKAIEKWYRVPFYFLKAKYLRYCLDEIKLTNHRIRWHFGFSKADLRKDSGIHYLHPKAKYLLNWVLKEELFESDCLNETVYTGINDPGLSSESMALRR |
| DN91527_c1_g1_i1 | 1,593 | 150 | 9.6 | 227 | 24.706 kDa | MHSFLAVHVGDARYHEGWLRAVYIAGADCRAAQSRHSFCCSGSCPPGWLLAAHPFGWLTDSMRCSTARSLLLSRLDGASSTVRALVVCLIFSWTSGSSLAAIVVLLGLLSSAVFVVPHLRLSSVNVFLCLPMLYFCWVYVSFSASLAAYRCRYSASSAVVHLAMLAPERLGFVPLGLASLSRPLYDHQVSKRTVLARVRQSRIACQLQHKHTDGRRGLNRSPPAGAT |
| DN92812_c0_g1_i4 | 873 | 249 | 29.2 | 182 | 19.770 kDa | MHSFLAVHVGDARYHEGWLRAVYIAGADCRAAQSRHSFCCSGSCPPGWLLAAHPFGWLTDSMRCSTARSLLLSRLDGASSTVRALVVCLIFSWTSGSSLAAIVVLLGLLSSAVFVVPHLRLSSVNVFLCLPMLYFCWVYVSFSASLAAYRCRYSASSAVVHLAMLAPERLGFVPLGLASLSRPLYDHQVSKRTVLARVRQSRIACQLQHKHTDGRRGLNRSPPAGAT |
| DN92884_c0_g1_i1 | 1,864 | 130 | 7 | 195 | 21.961 kDa | MLGVAGPVPCTDNSQPGSAATAAPSQRKSLLSSSYGPYYGCMRRRPTQQRLSHPARCRPCWAAGLLRVGGTTSPRPSHAAAAACSQQLVGGRRQRWRWHRAEHILARRRLVLLLLLGRADVNALLLAIFLARWPVLGGHPSPAAVFAAYDACERRIRPDTEPFMRLSPRCLCKPAAVATLAS |
| DN92884_c0_g1_i4 | 1,895 | 112 | 6 | 195 | 21.961 kDa | MDASHTWCSDTARGKCKWTAYLVLPSWSLPPANPPPRSRNEAYPNTVPLVLVCCHKGCRRRDAAVDTKCVSPSLHSVPLNDAMSLLCGDDRGAKVQAKGDRRHLPRPHKPRNSRHRKFAAAARALARQQRRVVENGFLICSGEATVQVLHVLPSRCYTKDRAVRAELRQRRRRQPHSGLRSPSTCSCTGWRRARQ |
| DN92884_c0_g1_i5 | 1,830 | 107 | 5.9 | 169 | 18.397 kDa | MDASHTWCSDTARGKCKWTAYLVLPSWSLPPANPPPRSRNEAYPNTVPLVLVCCHKGCRRRDAAVDTKCVSPSLHSVPLNDAMSLLCGDDRGAKVQAKGDRRHLPRPHKPRNSRHRKFAAAARALARQQRRVVENGFLICSGEATVQVLHVLPSRCYTKDRAVRAELRQRRRRQPHSGLRSPSTCSCTGWRRARQ |
| DN93420_c0_g1_i1 | 2,531 | 32,812 | 1332.5 | 801 | 93.586 kDa | MKIFVTSKVSNTQDILYWTQRRMETEKWDIDKNILLNVSLMLSIFALGLMFAVTNYIFIATGLIGKVWNLRQESKNVDLISFYACMVGTLSLSQHSLTMAPLLLLPLGIYSYRATLVPLVELVKVSSATSVLIMMVSTVVSHSFGNFRALQVSLLIYNISISDKCTTKDARYLLLYKMLLNMGATSPCLQKYLKSPFSYFKTTENSKCYHVEPCLGLRAKTRLPKKILTVVLLSKWMDSQVNKLAGLDPVMTLLFGWLHTLGFYPPFIKPIAGVVTAARGKRRANMDVEKLAEWAVNKQNYVKTHDGVIRTLFLLYRQRFDKMSKKERKLAYQFSNIPNFRLTKAKVNALKMKILKTSSYLPAVFDINYKPYTSMVKVRIEKEYIKTPLTKEAERAWSSFRGCYTDDTKRVLIKETVLTQSGSCFFKMKSDITKKENFKEMRKKARSLTFLHSTKTMMTRNTNCVYRRKFKMQKKTVQDADLKSTFNKKIDPSKLRHFTKPLMMTQKVTCENIDTMRKVDYFHASLNYKLQEQAYYCNSSPNMVCDALKFVEGIRNLQKQNIETPPIQDHHYSGYIAEITAAAEKKKKLIEDARAELVGPVTSQRAPKREKKEMRRSLRKERKKKYKEDQKKKKEEMDEYGYYSMDEEERYGMADEDDGEDKLTFEELDEMYREPELDETARFKPGEWQPPKHVPLPKPPKVTVEEYQFPARYKKIIRFKAHEAQWLVRRKNRFLEKLEDTELKKFAPQITNWVVNATRTRMMSLWGMTTFAKPRNTFQTDLKEMKARLRMIEVCRLGSKK |

^1^raw reads were mapped against the virus sequence using Geneious 10.2.6 assembler with medium-low sensitivity; ^2^mean value generated by Geneious 10.2.6; ^3^the length of the longest predicted protein; ^43^the predicted sequence of the longest protein

# Supplementary Figures


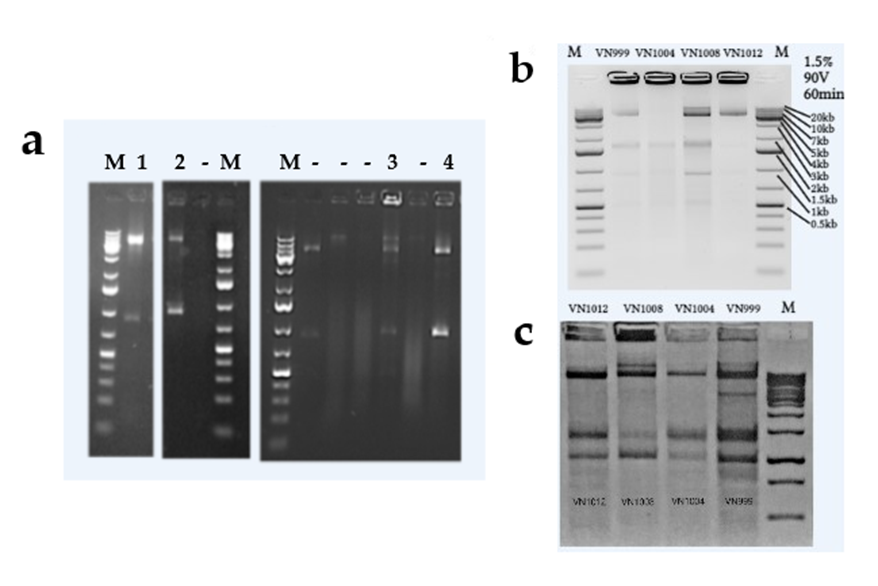


**Supplementary Figure 1.** (a), (b), (c) dsRNA banding pattern of *Phytophthora castaneae* isolates detected by three independent dsRNA extractions followed by agarose gel electrophoresis. (a) lane 1: VN999; lane 2: VN1004; lane 3: VN1008; lane 4: VN1012; isolates that are not part of this study are indicated by “-. “ (b) dsRNA banding patterns in all four isolates. (c) dsRNA banding patterns of all four isolates, where a band between 10-20 kb appears, plus an additional band of approximately 4 kb in VN999. As the samples were not treated with S1 nuclease, it cannot be ruled out that some of the bands, in particular those at ca. 1 and 1.5 kb, belong to oomycete host rRNA. M in (a) and (b) is DNA marker (GeneRuler 1 kb Plus DNA Ladder, 75–20,000 bp, Thermo Scientific, MA, USA). M in (c) is (GeneRuler 1 kb DNA Ladder, 250–10,000 bp, Thermo Scientific, MA, USA).

**
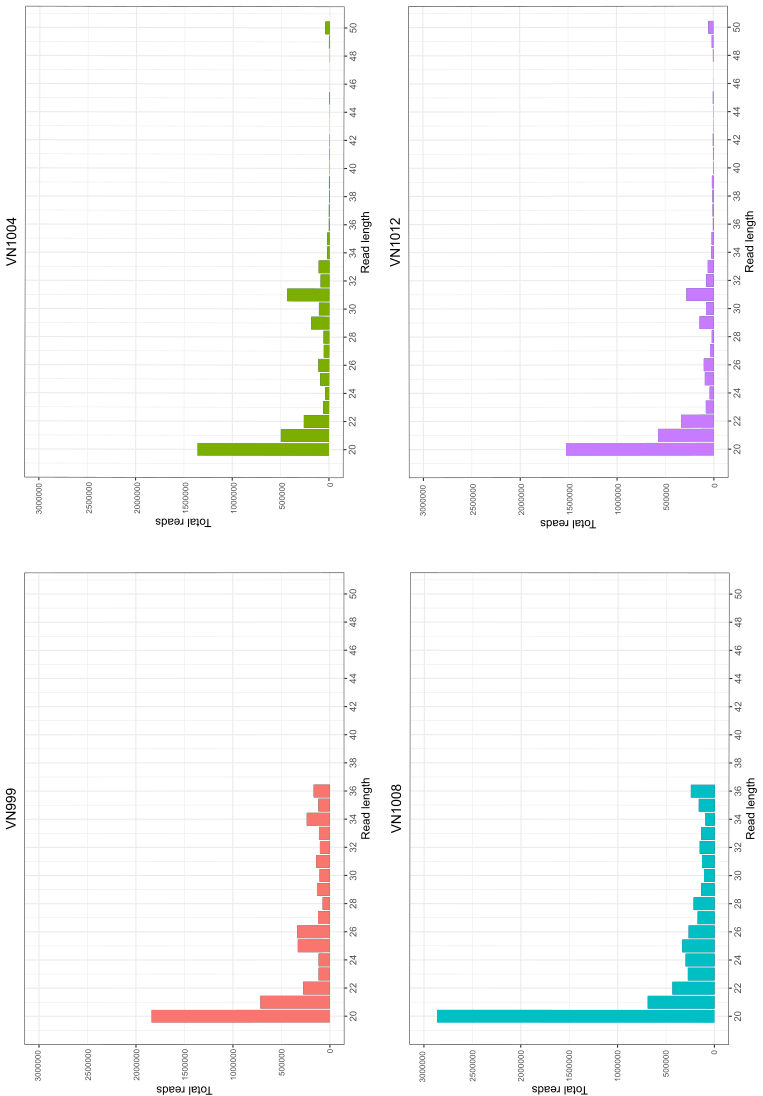
**

**Supplementary Figure 2**. Distribution of small RNA reads by size in *P. castaneae* isolates VN999, VN1004, VN1008 and VN1012. The final graphs were constructed in R studio v2022.02.0.


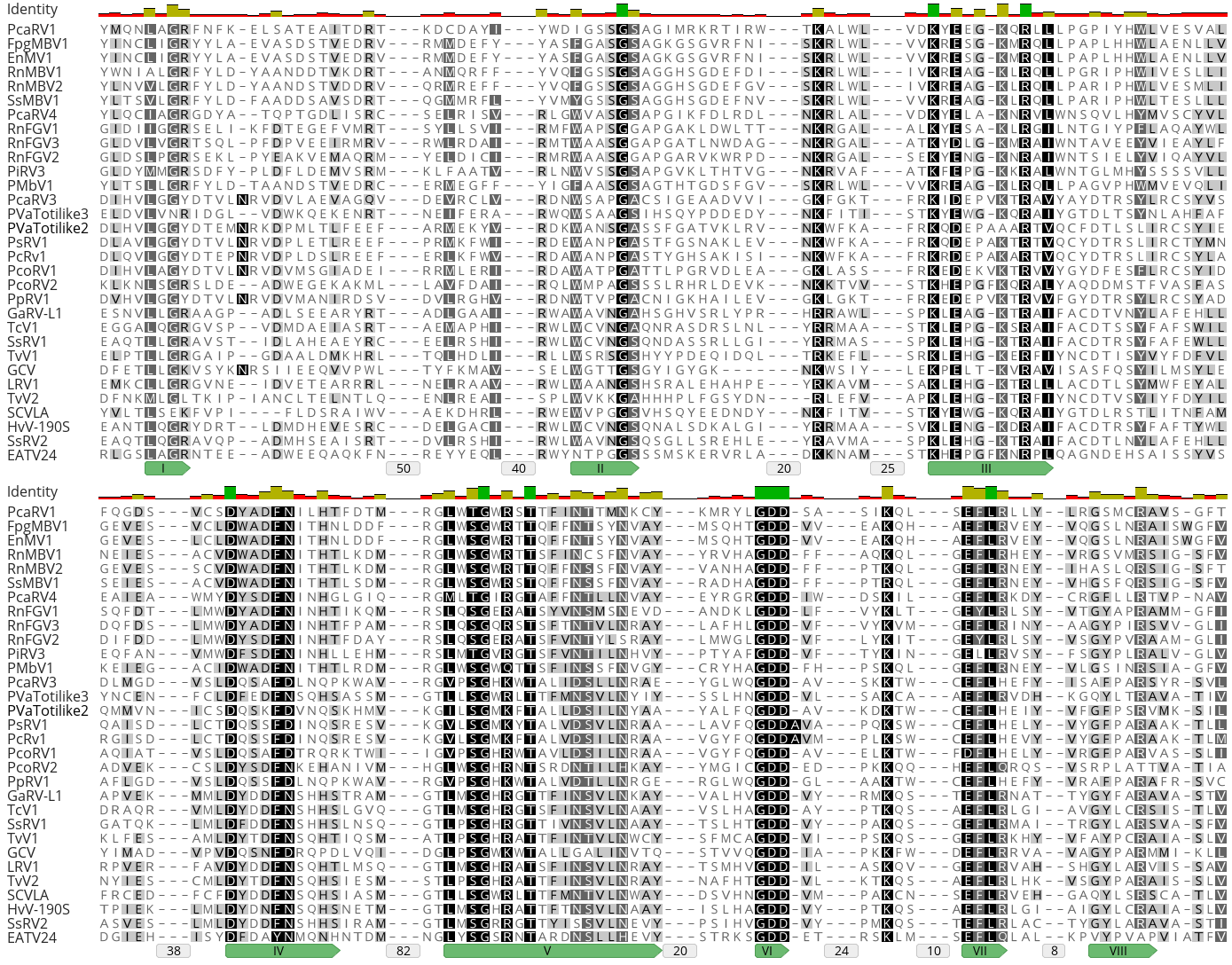


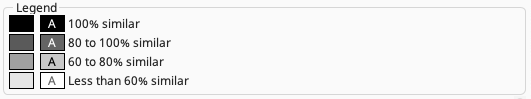


**Supplementary figure 3**. Amino acid alignment showing conserved motif (I-VIII) of RdRp of selected totiviruses, fusagraviruses and megabirnaviruses.The megabirnaviruses included in the alignment are: PcaRV1, Phytophthora castaneae RNA virus 1; FpgMBV1, Fusarium pseudograminearum megabirnavirus 1 (AYJ09269); EnMV1- Entoleuca megabirnavirus 1 (AVD68671); RnMBV1, Rosellinia necatrix megabirnavirus 1/W779 (YP_003288763); RnMBV2, Rosellinia necatrix megabirnavirus 2-W8 (YP_009227124); SsMBV1, Sclerotinia sclerotiorum megabirnavirus 1 (YP 009143529). The totiviruses included in the alignment were: PcaRV3, Phytophthora castaneae RNA virus 3; PVaTotilike3, Plasmopara viticola associated totivirus 3 (QGY72630), PVaTotilike2, Plasmopara viticola associated totivirus-like 2 (QGY72636), PsRV1, Pythium splendens RNA virus 1 CP-RdRp (BBJ21453), PcaRV1- Phytophthora cactorum RNA virus 1 CP-RdRp (QJS39952), PcoRV1- Phytophthora condilina RNA virus 1 (MW503714), PcoRV2, Phytophthora condillina RNA virus 2 (MW503715), PpRV1- Pythium polare RNA Virus 1 (YP_009552275); GaRV-L1, Gremmeniella abietina RNA virus L1 (NP_624332); TcV-1, Tolypocladium cylindrosporum virus 1 (YP_004089630); SsRV1, Sphaeropsis sapinea RNA virus 1 (NP_047558); TvV1-Trichomonas vaginalis virus 1 (NP_620730); GCV, Giardia canis virus (ABB36743); LRV1, Leishmania RNA virus 1 (NP_041191); TVV2, Trichomonas vaginalis virus 2; SCVLA- Saccharomyces cerevisiae virus L-A SCVLA (Q87022); HvV190S, Heminthosporium victoriae virus 190s; SsRV2, Sphaeropsis sapinea RNA virus 2; EATV24, Erysiphales associated totivirus 24 (QIP68042). The fusagraviruses included in the alignment were: PcaRV4, Phytophthora castaneae RNA virus 4; RnFGV1, Rosellinia necatrix fusagravirus 1 (BBB86778); RnFGV3, Rosellinia necatrix fusagravirus 3 (BBB86785); RnFGV2, Rosellinia necatrix fusagravirus 2 (BBB86783); PiRV3, Phytophthora infestans RNA virus 3 (YP 009551328); PMbV1, Pleosporales megabirnavirus 1 (ALO50147). Gray boxes with numbers represent the number of positions deleted in the MUSCLE alignment. The % of similarities are calculated based on Blosum62 score matrix with a threshold of 1.


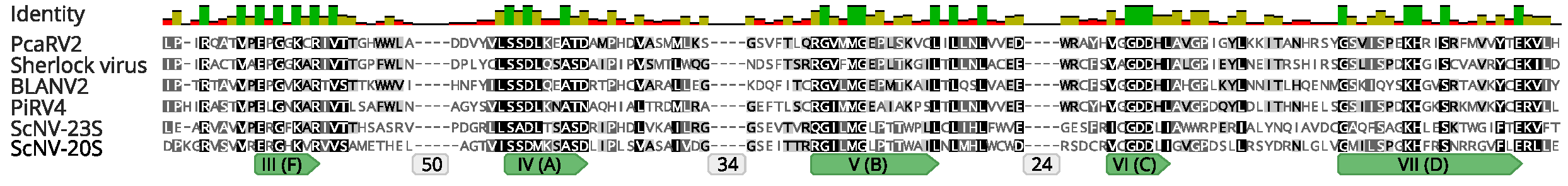


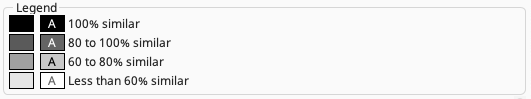


**Supplementary figure 4.** Conserved aa sequence motifs (III-VII) of RdRp of PcaRV2 and members of the family *Narnaviridae*. ScNV-23S, Saccharomyces 23S RNA narnavirus (NP660177), ScNV-20S, Saccharomyces 20S RNA narnavirus (NP660178); PiRV4, Phytophthora infestans RNA virus 4 (YP009241365), Sherlock virus (QED21500), and BLANV2, Bremia lactucae associated narnavirus 2 (QIP68013). Gray boxes with numbers represent the number of positions deleted in the MUSCLE alignment. The % of similarities are calculated based on Blosum62 score matrix with a threshold of 1.


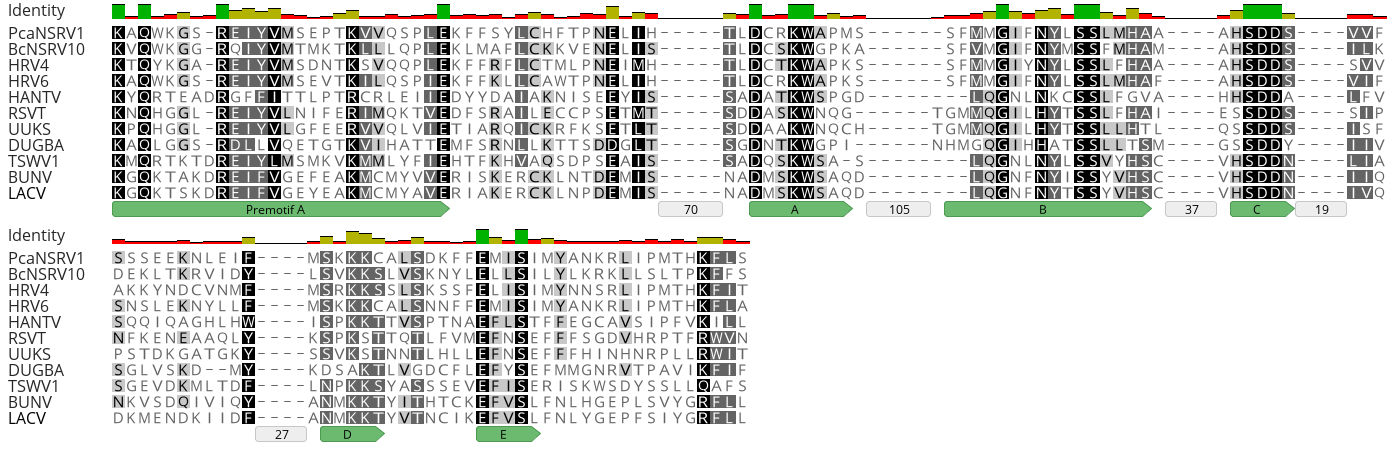


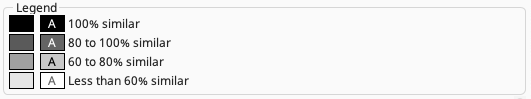


**Supplementary Figure 5**. Amino acid alignment showing Bunya RdRp conserved motifs A to E and premotif A within the RdRp of PcaNSRV1 and selected bunyaviruses. BcNSRV10, Botrytis cinerea negative stranded RNA virus 10; HRV4, Halophytophthora virus 4 (MT277353); HRV6, Halophytophthora virus 6 (MT277355); HANTV, Hantaan virus 76-118 (P23456); RSVT, Rice stripe virus (Q85431); UUKS, Uukuniemi virus S23 (P33453); DUGBA, Dugbe virus (accession number Q66431); TSWV1, Tomato spotted wilt virus (P28976); BUNV, Bunyamwera virus (P20470); LACV, La Crosse virus (2XI7_D) (Q8JPR2). The gray boxes with numbers represent the number of positions deleted in the MUSCLE alignment. The % of similarities are calculated based on Blosum62 score matrix with a threshold of 1.
